# Caffeine-Induced Premature Chromosome Condensation Results in the Apoptosis-Like Programmed Cell Death in Root Meristems of *Vicia faba*

**DOI:** 10.1371/journal.pone.0142307

**Published:** 2015-11-06

**Authors:** Dorota Rybaczek, Marcelina Weronika Musiałek, Aneta Balcerczyk

**Affiliations:** 1 Department of Cytophysiology, Institute of Experimental Biology, Faculty of Biology and Environmental Protection, University of Łódź, Łódź, Poland; 2 Department of Molecular Biophysics, Faculty of Biology and Environmental Protection, University of Łódź, Łódź, Poland; University of Vigo, SPAIN

## Abstract

We have demonstrated that the activation of apoptosis-like programmed cell death (AL-PCD) was a secondary result of caffeine (CF) induced premature chromosome condensation (PCC) in hydroxyurea-synchronized *Vicia faba* root meristem cells. Initiation of the apoptotic-like cell degradation pathway seemed to be the result of DNA damage generated by treatment with hydroxyurea (HU) [double-stranded breaks (DSBs) mostly] and co-treatment with HU/CF [single-stranded breaks (SSBs) mainly]. A single chromosome comet assay was successfully used to study different types of DNA damage (neutral variant–DSBs *versus* alkaline–DSBs or SSBs). The immunocytochemical detection of H2AXS139Ph and PARP-2 were used as markers for DSBs and SSBs, respectively. Acridine orange and ethidium bromide (AO/EB) were applied for quantitative immunofluorescence measurements of dead, dying and living cells. Apoptotic-type DNA fragmentation and positive TUNEL reaction finally proved that CF triggers AL-PCD in stressed *V*. *faba* root meristem cells. In addition, the results obtained under transmission electron microscopy (TEM) further revealed apoptotic-like features at the ultrastructural level of PCC-type cells: (i) extensive vacuolization; (ii) abnormal chromatin condensation, its marginalization and concomitant degradation; (iii) formation of autophagy-like vesicles (iv) protoplast shrinkage (v) fragmentation of cell nuclei and (vi) extensive degeneration of the cells. The results obtained have been discussed with respect to the vacuolar/autolytic type of plant-specific AL-PCD.

## Introduction

In order to preserve a specific cell number and maintain organism balance, cells are equipped with a genetically designed mechanism known as programmed cell death (PCD). It is a unique set of events that lead to controlled and organized destruction of redundant, damaged or nonfunctional cells [1–3]. PCD is a natural consequence of ageing, but it also may be switched on by either environmental stress factors or developmental irregularities. Cell dying is one of the most complicated processes to follow due to the diversity of stimuli that may influence it, as well as regulatory mechanisms responsible for cell destruction and final removal. PCD associated with cell differentiation is known as developmental cell death (DCD) [2,4].

Differencies regarding PCD that can be observed between animals and plants or even within these groups make description of this process difficult. Due to divergencies in biochemical mechanisms and morphological cell changes, there are three types of PCD distinguished in animals: apoptosis (Type I of PCD), autophagy (Type II od PCD) and necrosis; and two major types in plants: autolytic (vacuolar) and non-autolytic (necrotic), which differ in terms of cytoplasm destruction [5]. The main dispartity between plants and animals is the mechanism of cell debris removal following cell death. The existence of cell wall in plants makes phagocythosis impossible, therefore an additional process of apoptotic bodies degradation is needed for the adjacent cells to be able to start collecting the remains. The presence of vacuoles and additional organelles can also impact the process. Autolytic plant PCD is associated with hydrolases being released from a vacuole after its collapse, thus resulting in rapid clearance of the cytoplasm [6].

Attempts to unify PCD terminology concerning animal and plant cells has not been easy and is mainly limited by cell structure disparities. Detailed analysis has revealed an analogy between necrosis and non-autolytic plant PCD. It has also been confirmed that some symptoms of autophagy in animal cells are identical with those of autolytic plant PCD. The biggest controversies arouse over apoptosis, as until recently it was believed to be absent from plants. Due to the fact that some specific symptoms have also been observed in plants, the term apoptosis-like programmed cell death (AL-PCD) has been introduced [3,7–8]. Research clearly shows that AL-PCD is an integral part of plant ontogenesis controlled by cellular oxidative state, phytohormones, and DNA methylation. Ultrastructural changes observed in a plant cell during AL-PCD are classified as follows: (i) compaction and vacuolization of the cytoplasm, (ii) specific fragmentation of the cytoplasm and unique single-membrane vesicles containing the active organelles in a vacuole, (iii) intensive synthesis of mitochondrial DNA in vacuolar vesicles, (iv) cessation of nuclear DNA synthesis, (v) condensation and marginalization of chromatin inside the nucleus, and (vi) internucleosomal fragmentation of nuclear DNA [9].

Apoptotic events of chromatin condensation, as described in detail by Banfalvi et al. [10] in Chinese hamster ovary (CHO) cells, were compared with the phenomenon of premature mitosis or mitotic catastrophe [11–13]. Premature mitosis (premature chromosome condensation, PCC) results in prematurely condensed chromosomes, aberrant mitosis, followed by cell death [11]. PCC is also indicated as a chromosome condensation before DNA doubling has been finished in the S-phase [14]. PCC may be induced by many factors (i.e. mutations, cell fusion, chemical agents, etc.). Some PCC inducers, such as caffeine, okadaic acid, staurosporine, calyculin A, were also found to trigger apoptosis at higher concentrations [11,15]. However, spindle formation, p34^cdc2^ activation and phosphorylation of histones H1 and H3 occurred only in PCC, which indicates molecular differences between these processes (i.e. PCC *versus* apoptosis [11–12,16]).

The activation of PCD-related pathways leads to a set of changes described well in literature [8,17]. PCD is defined as an active process leading to the elimination of cell(s) that is designed to maintain homeostasis, ensure proper growth and enable further development of the organism [18]. However, it is indicated that the death of individual cells due to PCD may be also associated with simultaneous activation of a mechanism or mechanisms leading to the development of adaptive responses to stressful environmental conditions. Although PCD is often described and characterized in plant cell cultures, due to their uniformity, accessibility and reduced complexity which allows obtaining more general and sometimes more complete view on the process [3], our research is based on plant tissues. In the present article we focus on genotoxicity and visualization of symptoms of AL-PCD in whole plant tissues. This paper presents the collective results of many years of observing that a portion of the nuclei induced to PCC *via* caffeine-treatment (under conditions of permanent replication stress) enter the cell death pathway. The classification of various death types introduced by van Doorn in 2011 [5], and successive works of the Nomenclature Committee on Cell Death (NCCD), also taking into consideration a systematization of knowledge within this scope [19,20], have become for us the basis to put forward a conclusion on the occurrence of an AL-PCD in broad bean cells. *Vicia faba* root meristem cells used as a model system were treated with 2.5 mM hydroxyurea (HU) and after PCC induction by 5 mM caffeine (CF), were analyzed using the following methods: (1) double acridine orange (AO) and ethidium bromide (EB) staining (AO/EB); (2) comet assay, in an alkaline variant (used for detection of single-strand breaks [SSBs] within DNA) as well as in a neutral variant (used for detection of double-strand breaks [DSBs]); (3) TUNEL-assay; (4) standard immunocytochemistry; (5) tissue printing; (6) DNA ladder electrophoresis; (7) Western blot, and (8) transmission electron microscopy (TEM). We describe the different types of DNA damage and the early-to-late symptoms of AL-PCD at histochemical, immunocytochemical, biochemical as well as TEM levels. Finally, we also show that AL-PCD was not observed in *V*. *faba* root meristem cells after treatment with HU alone (i.e. under replication stress) but was visible after treatment with a mixture of HU/CF (i.e. after PCC induced *via* CF under conditions of prolonged replication stress). We show the presence of cell death-related symptoms in the plant cells thus proving a specific type of PCD. For this reason possible connotations with vacuolar/autolytic PCD are discussed.

## Materials and Methods

### Chemicals and antibodies

Hydroxyurea (HU, 2.5 mM), pararosaniline, bovine serum albumin (BSA), propidium iodide (PI) and 4',6-diamidino-2-phenylindole (DAPI) were purchased from Sigma. Caffeine (CF, 5 mM) was supplied by Merck, Triton X-100 by Fluka, RNase from SERVA. Other chemicals were obtained from POCH S.A. (if not indicated otherwise in the text).

Immunocytochemical and biochemical detection of PARP-2 was performed using rabbit polyclonal antibodies from Agrisera (Vännas, Sweden; #AS10675). The rabbit monoclonal antibodies specific to phospho-H2AX (Ser139) (20E3) were supplied by Cell Signaling (Danvers, MA, USA; #9718). Bound primary antibodies in all investigated cases were detected with the secondary goat anti-rabbit IgG AlexaFluor^®^488 antibody (Agrisera, Vännas, Sweden; ABIN2176504, for immunocytochemistry) and also the secondary anti-rabbit IgG (AP-linked) antibody (Cell Signaling, Danvers, MA, USA; 7054, for immunoblotting and tissue printing). The mouse monoclonal antibody β-actin (A5441) and the secondary goat anti-mouse (AP-linked; A3562) antibody were from Sigma-Aldrich (Saint Quentin, France).

### Plant material, growth conditions, HU-treatment and PCC induction

Seeds of *Vicia faba* var. *minor* cv. Nadwiślański (Center for Seed Production, Sobiejuchy, Poland) were dark-germinated at room temperature on wet filter paper in Petri dishes. Four days after imbibition, 3 cm seedlings were selected and incubated in (i) water (32 h; negative control); (ii) HU (2.5 mM for 32 h; S-phase synchronization; positive control), or (iii) 2.5 mM HU for 24 h and then transferred into a mixture of 2.5 mM HU and 5 mM caffeine for 8 h (HU/CF; total incubation time: 32 h; PCC induction), as described by Rybaczek [21]. During germination and incubation the roots were oriented horizontally and aerated continuously by gentle rotation of fluids in a water-bath shaker (30 rpm).

### Cytology

1.5-cm-long apical fragments of *V*. *faba* primary roots (n = 30 for each series) were fixed in cold Clarke’s mixture (absolute ethanol/glacial acetic acid; 3:1, *v/v*) for 1 h (according to Bruni et al. [22]), washed three times with 96% ethanol/rehydrated (70–30% ethanol) distilled water and subjected to Feulgen staining (according to Rybaczek et al. [23]). For this procedure, the roots were hydrolyzed in 4 M HCl (at room temperature for 2 h), and stained with Schiff’s reagent (pararosaniline). After staining (1 h), root fragments were rinsed three times in SO_2_-water and once in distilled water. 1.5-mm-long root sections were cut off and squashed in a drop of 45% acetic acid onto Super-Frost microscope slides (Menzel-Gläser) using the dry ice method. After removing the coverslips, the slides were dehydrated, air dried, and embedded in Canada balsam (Merck, Germany). The quantification of mitotic/PCC/AL-PCD cells and scoring of the micronucleus frequency (MN-test) were determined by counterstaining with Schiff's reagent for 1 h at room temperature. Three parameters were evaluated: (1) mitotic index (i.e. percentage of mitotic cells), (2) PCC index (i.e. percentage of PCC-type mitoses in relation to all mitoses, with the proviso that PCC-type aberrant mitoses were calculated as a sum: S-PCC + G2-PCC + segregation defects) and (3) AL-PCD index (i.e. percentage of Feulgen-stained nuclei showing signs of AL-PCD in relation to all meristem cells, i.e. either interphase or mitotic). The percentages were calculated based on 5,000 cells per treatment (1,000 cells on each of the 5 preparations in each series). The experiments were done in triplicate. Cytological observations were made using an Optiphot-2 microscope (Nikon) and images were recorded with a DXM 1200 CCD camera (Nikon). Macroscopic observations of roots (control *vs* treated with HU *vs* treated with HU/CF during PCC induction) were made using Stemi 2000C stereoscopic microscope (Zeiss, Jena, Germany) and images were recorded by AxioCam ERc5s CCD camera (Zeiss, Jena, Germany). Quantitative analyses were performed using AxioVision software, 4.8 version (Zeiss, Jena, Germany). Image processing was done in Adobe Photoshop 7.0 (Adobe Systems) or *ImageJ* 1.37c (Public Domain by Wayne Rasband) according to Abràmoff et al. [24].

### Estimation of cell death *in planta*: acridine orange and ethidium bromide staining

Fluorescence staining with acridine orange (AO) and ethidium bromide (EB) was used for detection of cell death according to the method described by Byczkowska et al. [8]. This method allows gradual staining of cells depending on their stage: living to dead. AO penetrates all cells both living and dead but EB can only enter a cell after disintegration of the cell’s membrane. Therefore, living cells containing only AO appear green under fluorescent microscopy, cells in early apoptosis appear green-yellow to yellow, cells in late apoptosis appear yellow-orange to bright orange, and dead cells appear as dark orange to bright red [8]. Briefly, 1.5-cm-long apical fragments of living roots (n = 30 for each series) were cut off and washed 2 times in 0.01M phosphate buffer, pH 7.4 (PHB) and stained for 4 min with 1 ml of a mixture containing 100 μg ml^-1^ AO and 100 μg ml^-1^ EB in PHB. After removing the 'staining mixture' the root fragments were washed 2 times in PHB, fixed with 1% glutardialdehyde (Merck) in PHB for 15 min, and cut with a razor blade along the long axis of the root. The thin sections obtained were washed 2 times for 2 min with PHB and placed onto Super Frost glass slides (Menzel-Gläser) with a drop of PHB. Observations were performed by Optiphot-2 fluorescence microscope (Nikon) equipped with a B-2A filter (blue light; λ = 495 nm). It is worth noting that the images typical of double AO/EB staining described here (distinguishable as green-yellow, yellow, yellow-orange, bright orange and red fluorescence; per Byczkowska et al. [8]) were only visible through a narrow-strip type of fluorescent filter (in a wide-strip all observed nuclei were totally green). Images were recorded at exactly the same time of integration using a DS-Fi1 CCD camera (Nikon, Japan) and Act-1 software (Precoptic, Warsaw, Poland). Quantitative analysis was performed using the basic functions of *ImageJ* v1.37c software (Public Domain by Wayne Rasband). To this end, we used the selection tool to trace out the area of each root which were then measured in pixels. Then, we used the cut-off threshold (threshold → thresholding method: minimum; cut off color: 125–255): (1) we selected all the points with the following colors: red, yellow and green, and (2) measured their surface areas using analyses in Excel (Microsoft Office, 2003). The whole root area was 100% and respective areas occupied by the three different colors in each of the roots were calculated (indicating green–living cells, yellow–dying cells; red–dead cells). In the next stage, analyses were performed in a similar manner, except that the analyzed areas were limited to each root zone (II—meristematic zone containing apical meristem, III—elongation zone, IV—differentiation zone). Means were calculated based on 300 cells per sample taken from three independent experiments.

### Single-cell microgel electrophoresis: alkaline *versus* neutral comet assay

The first step of the comet assay procedure consisted in the preparation of agarose-embedded *V*. *faba* protoplasts according to the method described by Tegeder et al. [25] with minor modifications. Briefly, 5-mm sections of root tips (n = 30 for each series) were incubated for 4 h in 10 mM citrate buffer (pH 4.8, 37°C) supplemented with 5% (w/v) cellulase R10, 1% (w/v) pectinase, 1% (w/v) macerozyme R-10 from *Rhizopus sp*. (Serva), 1% (w/v) hemicellulase from *Aspergillus niger* (Sigma) and 2.5% (w/v) pectolyase. The macerat was filtered through a ø 48 μm nylon mesh. The protoplast suspension was centrifuged at 100 G for 5 min at 4°C. The pellet was washed 2 times with the mannitol/MES buffer (0.5 M mannitol and 20 mM MES, pH 5.5). The sediment was suspended in a small amount of 0.5 M mannitol in the same buffer.

SSBs and DSBs were assessed with alkaline and neutral single-cell microgel electrophoresis (comet assay), respectively according to the method described by Potocki et al. [26]. Briefly, protoplasts originating from *V*. *faba* root tips were mixed with 0.7% low melting (LM) agarose, added to agarose-LM slides, lyzed overnight with proteinase K (0.5 mg ml^-1^) and reduced glutathione (2 mg ml^-1^) in a lysis solution (1.25 M NaCl, 50 mM EDTA, 100 mM Tris-HCl, 0.01% N-lauroylsarcosine sodium salt, pH 10) for 2 h at 37°C. The slides were then placed in a horizontal gel electrophoresis unit (Bio-Rad). The neutral comet assay buffer was as follows: 100 mM Tris-HCl, 0.5 M NaCl, 1 mM EDTA, 0.2% DMSO, pH 10; and the alkaline comet assay buffer was as follows: 1 mM EDTA, 0.2% DMSO, 300 mM NaOH, pH >12. Next, the slides were stained with 0.25 μM YOYO-1 (Invitrogen Corporation, Grand Island, NY, USA) in 2.5% DMSO and 0.5% sucrose, mounted with a coverslip and stained with DAPI (0.4 μg ml^-1^; Sigma-Aldrich). Observations were made immediately using an AxioImagerA1 fluorescence microscope (Zeiss, Jena, Germany) equipped with a blue light filter (excitation 470/40 nm; emission 525/50 nm) for YOYO-1, and a UV filter (UV light; excitation 365 nm; emission 445/50 nm) for DAPI. All images were recorded at exactly the same time of integration using an AxioCam ERc5s CCD camera (Zeiss, Jena, Germany) and AxioVision 4.8 software (Zeiss, Jena, Germany). Comet analyses were carried out automatically using OpenComet *ImageJ* software. Histograms were transferred manually to a diagram using Photoshop CS5 (Adobe Systems). The tail moment (TM) was calculated for every image of nuclei as the product of tail DNA (i.e. amount of DNA in the comet tail) times tail length (i.e. length of the comet tail measured from right border of head area to end of tail, according to the method described by Olive et al. [27] and Kołodziejek et al. [28]). We analyzed 30 images of nuclei from each experimental series. All experiments were repeated five times. Statistical analysis was carried out using Excel 2003.

### Terminal deoxynucleotidyl transferase-mediated dUTP (2'-deoxyuridine, 5'-triphosphate) nick end-labeling (TUNEL) assay in Click-chemistry technology

For detection of apoptosis TUNEL-assay (Click-iT^®^ TUNEL Alexa Fluor^®^ 594 Imaging Assay for microscopy and HCS, Invitrogen-Life Technologies, #C10246) was performed according to the protocol provided by the manufacturer (using slightly modified methods described by Jones et al. [29] and Gladish et al. [30]). In this assay, free 3'-OH ends of fragmented DNA were enzymatically labeled with fluorescein-modified nucleotide (i.e. fluorescein-dUTP) using terminal deoxynucleotidyl transferase (TdT). The Click-iT^®^ TUNEL Alexa Fluor^®^ 594 Imaging Assay was able to detect apoptotic cells of *V*. *faba* under the investigated conditions. Additionally for each batch, a negative control without the addition of TdT enzyme and a positive control with DNase I treatment, to generate strand breaks, were always included to ensure the reproducibility of the assay. The percentage of positive-labeled cells (red fluorescence typical of Alexa Fluor^®^ 594 azide: 590/615 nm) represents averages from three repetitions.

For each sample, 5-mm sections of root tips (n = 30 for each series) were fixed using 4% paraformaldehyde (Polysciences, #18814) in PBS for 45 min at room temperature. The fixation step was followed by a permeabilization step with 0.25% Triton X-100 in PBS for 20 min at room temperature. Next, terminal deoxynucleotidyl transferase-mediated dUTP (2'-deoxyuridine, 5'-triphosphate) nick end-labeling (TUNEL) assay was performed following the manufacturer's instructions strictly (Click-iT^®^ TUNEL Alexa Fluor^®^ Imaging Assay Protocol) and the nuclei were stained for 3 min with 0.3 mg ml^-1^ 4',6-diamidino-2-phenylindole (DAPI). Finally, the cells were mounted in Vectashield embedding medium (Vector Laboratories, CA, USA). Images were collected with an AxioImagerA1 fluorescence microscope (Zeiss, Jena, Germany) equipped with a green light filter (excitation 545/25 nm; emission 605/70 nm) for AlexaFluor^®^594, and a UV filter (UV light; excitation 365 nm; emission 445/50 nm) for DAPI. All images were recorded at exactly the same time of integration using an AxioCam ERc5s CCD camera (Zeiss, Jena, Germany) and AxioVision 4.8 software (Zeiss, Jena, Germany). Image processing was done in Adobe Photoshop 7.0 (Adobe Systems).

### DNA extraction and separation

1.5-mm-long sections of roots (n = 30 roots for each series; repeated twice) were homogenized in a mortar according to the method described by Byczkowska et al. [8] using 600 μl extraction buffer (2% SDS; 0.5 M NaCl; 100 mM Tris-HCl, pH 8.0; and 50 mM EDTA, pH 8.0) for 60 s. The homogenates were incubated at 65°C for 40 min, vortexed, chilled on ice for 10 min and centrifuged (12,000 G, 10 min, 4°C). Then, chloroform/isoamyl alcohol (24:1) was added (1.0 volume per each sample). Next, the samples were vigorously mixed (by inversion) for 2 min and centrifuged at 12,000 G for 1 min at 4°C. The supernatant was transferred to a fresh Eppendorf tube and extracted with 0.8 volume of cold isopropanol for 2 min. 500 μl of 70% ethanol was added to the pellet, microcentrifuged for 2 min (minispin, Eppendorf), dried and re-suspended with 40 μl of TE buffer (10 mM Tris HCl, 1 mM EDTA, pH 8.0; BioShop^®^ Canada Inc., Burlington) containing RNase A (20 μg ml^-1^). Isolated samples of DNA were dissolved in distilled nuclease-free water, and separated on 1.5% agarose gel with 0.5 μg ml^-1^ ethidium bromide. As a DNA marker, 1 kb DNA ladder was used, 250–10,000 bp (Fermentas, Thermo Fisher Scientific). The separated DNA samples were visualized under UV light.

### Total protein extraction and Western blotting

Proteins were extracted from 1.5-mm-long sections of roots (n = 30 roots for each series; repeated twice) using TriPure Isolation Reagent (Roche Diagnostics Corporation, Indianapolis, IN, USA) according to the instructions of the manufacturer. Total protein concentrations in the cell lysates were determined using an Ultrospec 110pro (Amersham Biosciences, Austria). Total protein extracts were fractionated on 4–12% polyacrylamide-SDS gel and blotted onto nitrocellulose membrane (Ø 0.45 μm, Schleicher & Schüell, Germany). Signals were visualized with NBT/BCIP (Nitro blue tetrazolium chloride/5-bromo-4-chloro-3-indolyl phosphate, toluidine salt, Sigma-Aldrich, Saint Quentin, France) as substrates. Actin protein was used as an internal control (according to Rybaczek and Kowalewicz-Kulbat [14]).

### Tissue printing

1.5-cm-long apical fragments of roots (n = 15 roots for each series; repeated twice) were dissected longitudinally and transversely (cross-section at the level of the meristematic zone) and blotted onto a nitrocellulose membrane according to the method described by Cassab [31]. The following primary antibodies were used: (1) anti-H2AXS139Ph; (2) anti-PARP-2, as well as secondary antibodies conjugated to alkaline phosphatase. The color reaction was induced (for 10 min) using substrates for alkaline phosphatase (nitroblue tetrazolium; NBT and 5-bromo-4-chloro-3-indolyl phosphate; BCIP) in a buffer containing: 100 mM Tris, pH 9.5; 100 mM NaCl; 5 mM MgCl_2_.

Root tissue prints were made using a Stemi 2000C stereoscopic microscope (Zeiss, Jena, Germany) and images were recorded on an AxioCam ERc5s CCD camera (Zeiss, Jena, Germany). Image processing was done in Adobe Photoshop 7.0 (Adobe Systems).

### Immunocytochemistry

Immunocytochemical assays were performed according to the method described earlier (Rybaczek and Maszewski [32] and Rybaczek et al. [33]). Excised apical parts of *V*. *faba* roots (1.5-mm-long sections; n = 30 for each series) were fixed for 45 min (18°C) in PBS-buffered 3.7% paraformaldehyde, washed several times with PBS and placed in a citric acid buffered digestion solution (pH 5.0; 37°C for 45 min) containing 2.5% pectinase (Fluka), 2.5% cellulase (Onozuka R-10; Serva) and 2.5% pectolyase (ICN). After removing the digestion solution, the root tips were washed 3 times in PBS, rinsed with distilled water and squashed onto Super Frost Plus glass slides (Menzel-Gläser). The air-dried slides were pretreated with PBS-buffered 5% BSA at 20°C for 50 min and incubated overnight in a humidified atmosphere (4°C) with a primary antibody (raised against proteins indicated in the subsection *Chemicals and antibodies*) dissolved in PBS containing 1% BSA (at a dilution of 1:100). Following incubation, the slides were washed 3 times in PBS and incubated for 1 h (18°C) with Agrisera secondary goat anti-rabbit IgG DyLight^®^488 antibody (AS09 633; 1:1000). Nuclear DNA was stained with 4’,6-diamidino-2-phenyl-indole (DAPI, 0.4 μg ml^-1^; Sigma-Aldrich). Following washing with PBS, the slides were air dried and embedded in Vectashield mounting media for fluorescence assessment (Vector Laboratories). The labeling index (LI) was calculated as the ratio of immunofluorescence-positive cells to all the cells in a meristematic population. Observations were made using an AxioImagerA1 fluorescence microscope (Zeiss, Jena, Germany) equipped with a blue light filter (excitation 470/40 nm; emission 525/50 nm) for AlexaFluor^®^488-conjugated antibodies, a green light filter (excitation 545/25 nm; emission 605/70 nm) for AlexaFluor^®^555-conjugated antibodies, and a UV filter (UV light; excitation 365 nm; emission 445/50 nm) for DAPI. All images were recorded at exactly the same time of integration using an AxioCam ERc5s CCD camera (Zeiss, Jena, Germany) and AxioVision 4.8 software (Zeiss, Jena, Germany). Fluorescence signals were computed using *ImageJ* 1.37c software (Public Domain by Wayne Rasband) according to Abràmoff et al. [24]. Analysis was performed based on 500 cells (100 cells per each preparation) for each experimental series. The experiments were done in triplicate.

### Transmission electron microscopy (TEM)

TEM was used to examine the morphology of nuclei from untreated *V*. *faba* root meristem cells and the cells treated with HU or the mixture of HU/CF. The apical parts of roots (1.5-mm-long sections, n = 30 for each series) were fixed in 2% glutaraldehyde in 1% cacodylate buffer (pH 7.3) for 3 h at 4°C, post-fixed in 1% osmium tetraoxide in the same buffer for 3 h, and dehydrated in an ascending ethanol series. After infiltration with a medium consisting of Epon 812 and Spurr’s resin, ultrathin sections were double stained with uranyl acetate and lead citrate according to Reynolds [34]. The sections were examined and photographed in a JEM-1010 transmission electron microscope (JEOL, Ltd.). Observations were based on ultramicroscopic photographs taken of at least 50 cell nuclei. The experiments were done in triplicate (i.e. the whole TEM-related procedure was performed three times).

### Statistical analysis

Statistical analyses were performed with STATISTICA 8.0 PL software (StatSoft INC, Tulsa, Oklahoma). All of the experiments were done at least in triplicate. All data were expressed as mean ± SD. Differences between groups were assessed by the non-parametric Mann–Whitney *U* test (for impaired data). Student's *t*-test was used for data normally distributed. *P* ≤ 0.05 was considered significant according to Iglesias-Guimarais et al. [35]. Means values of the number of micronucleus (MN) per 1,000 cells were calculated for significance among all experimental series tested and the DMSO control. Comet assay-related data were assessed by ANOVA and Tukey's *a posteriori* test. All statistical calculations were performed with the sequence of actions typical for statistical analyses: (1) verification of data distribution, (2) verification of homogeneity of variance, (3) evaluation of differences between the examined objects, followed by (4) analysis of differences using *t* Student, Tukey or Mann-Whitney *U* tests. The incidence of an association was investigated: (1) between the control and the HU; (2) between the control and the HU→HU/CF (i.e. PCC); as well as (3) between the HU and the HU→HU/CF (i.e. PCC).

## Results

### Co-treatment with HU/CF triggers either premature chromosome condensation (PCC) or apoptosis like-programmed cell death (AL-PCD) in *V*. *faba* root meristem cells

First, *V*. *faba* root meristems were used as a model system to study whether CF had a role in the induction of AL-PCD in HU-synchronized and next HU/CF co-treated cells. The final concentrations of the agents (2.5 mM HU and 5 mM CF) were chosen in a series of preliminary tests using different chemicals, various doses, different protocols and various durations of treatment [21,33,36]. Cytological symptoms of PCC comprise aberrant mitoses with (i) irregular condensation of chromatin in prophase, (ii) chromosome fragmentation, (iii) disturbances in the chromosome's metaphase system; (iv) lost and lagged chromatids and chromosomes during anaphase, and (v) segregation defects e.g. chromosomal bridges (S1 Fig). Differentiation between particular phenotypes (S-PCC *versus* G2-PCC) was based on the fragmentation degree of chromosomes forced to undergo premature mitosis. Numerous fragmentations without chromatid-like pair elements indicated S-PCC phenotype (S1 Fig), while a relatively small number of breakpoints i.e. <20, leading to the loss of relatively large fragments of chromosomes during anaphase were characteristic of the cells showing G2-PCC phenotype (S1 Fig).

In the negative control series (32-h incubation in water) we observed 11.7% ± 1.5 of mitotic cells (all of them with a correct morphology; S1A and S1A'' Fig). In the cells treated with HU for 32 h (S1B and S1B' Fig), the mitotic index decreased to 2.1% ± 0.8, and chromosomes showing some aberrations appeared (6.1% ± 0.4 population of mitotic cells within the HU-treated series; S1B'' Fig). The cells subjected to 24-h blocking in 2.5 mM HU and then transferred into HU/CF mixture showed PCC symptoms in 60.9% ± 2.4 cells derived from the population of dividing cells (estimated as 10.8% ± 1.3; S1C'' Fig). The value 10.8% is the sum of cells showing a set of abnormalities shared by both phenotypes typical of PCC-type aberrant mitoses [i.e. S-PCC (8.0% ± 0.9) and G2-PCC (1.9% ± 0.4)] as well as small fraction of cells showing normal succession of chromosomal events despite of HU/CF co-treatment (i.e. 0.9% ± 0.3). Differences in the percentage of *V*. *faba* cells during consecutive stages of mitosis or PCC were significant (p ≤ 0.01). An association was found between the control and HU, as well as between the control and PCC (i.e. HU/CF co-treatment in HU-synchronized cells).

Quantitative analysis revealed that AL-PCD cells (i.e. cells containing a nucleus with extremely strongly condensed chromatin) were only observed in the HU/CF co-treated series (5.3% ± 1.1; S1E Fig) and not detected in either the control (32-h water-incubated; negative control) or the HU-treated cells (32-h; positive control). Additionally, in all the experimental series tested (S1A' Fig, S1B' Fig and S1C' Fig), the MN-test was used to determine the frequencies of micronuclei in interphase *V*. *faba* cells. The number of micronuclei per 1,000 cells were 0.6 ± 0.4, 2.1 ± 0.9, 6.9 ± 1.7, for the control, HU and HU/CF series respectively (S1D Fig). All the correlations related to the MN-test were significant. An association was found between the control and HU (p ≤ 0.05), as well as between the control and PCC (i.e. HU/CF co-treatment in HU-synchronized cells, p < 0.001).

The results obtained seemed to support our preliminary idea that in some populations of meristematic cells in which PCC was forced by CF, the induction of AL-PCD resulted from the aberrant course of premature mitoses, while the appearance of MN additionally indicated disturbances in the division of the genetic material into two opposite poles in the cell.

### The induction of PCC is crucial for the generation of DNA damage. HU mainly induced double-stranded breaks (DSBs) and HU/CF co-treatment induced single-stranded breaks (SSBs)

**Fig 1:**
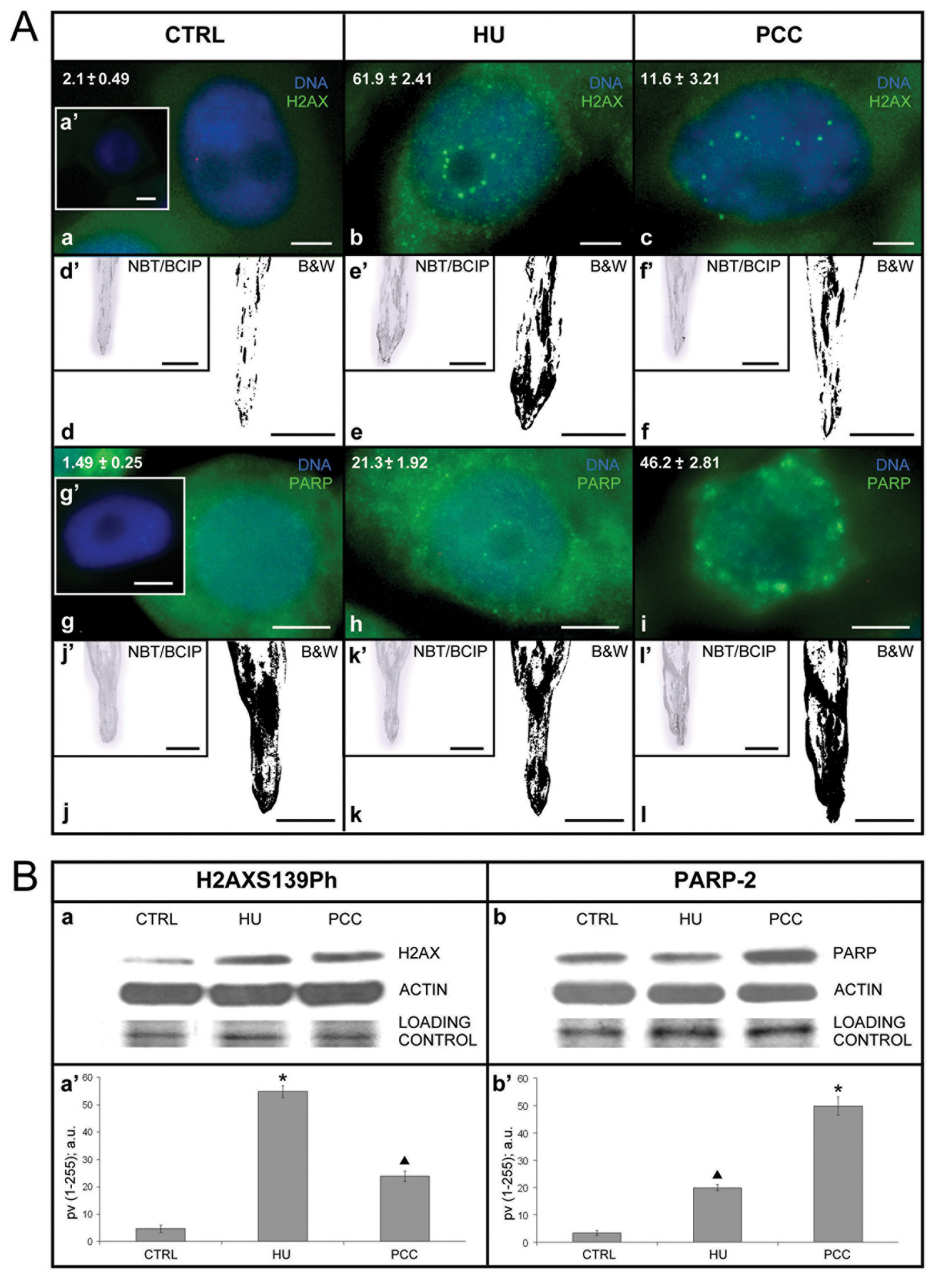
Histone H2AX phosphorylated at S139 (H2AXS139Ph) is a marker of double-stranded breaks (DSBs), while poly(ADP-ribose) polymerase-2 (PARP-2) is a marker of single-stranded breaks (SSBs). (A) Results of the immunocytochemical analysis and the method of *tissue printing*. (a-a', b-c) the presentation of superimposed fluorescence images (DAPI-related in blue and H2AXS139Ph-related in green) after the immunocytochemical detection of H2AXS139Ph in (a) control, (b) after 2.5 mM hydroxyurea-treatment (HU) for 32 h, (c) after 24-h synchronization under the influence of 2.5 mM HU and 8-h co-treatment with 2.5 mM HU and 5 mM caffeine (CF). (a') negative control; incubation exclusively with secondary antibodies. The values of marking indices (expressed in percents) are presented in the top left corner on the following images: (a)–for control series; (b)–after 32-h treatment with HU; (c)–after the induction of premature chromosome condensation (PCC) under the influence of HU/CF. *Scale bars* in a-a', b-c are 20 μm. (d-f) identification of H2AXS139Ph in the top sections of *Vicia faba* roots by the method of *tissue printing*, negative images. In the top left corner of each negative image, there is a miniature of the same fragment of nitrocellulose membrane in color, i.e. stained in the reaction of NBT/BCIP (d'-f'). (d-d') control, (e-e') HU, 32 h, (f-f') HU for 24 h and co-incubation HU/CF for 8 h (total incubation time: 32 h). *Scale bars* in d'-f' and d-f are 10 mm. (g-g', h-i) presentation of superimposed fluorescence images (DAPI-related in blue and PARP-2-related in green) after the immunocytochemical detection of PARP-2: (g) control, (h) after HU-treatment for 32 h, (i) after 24-h synchronization under the influence HU and 8-h co-treatment with HU/CF. (g') negative control; incubation exclusively with secondary antibodies. The values of marking (expressed in percents) are presented in the top left corner of the following images (g) control series; (h) after 32-h treatment with HU; (i) after the induction of PCC under the influence of HU/CF. *Scale bars* in g-g', h-i are 20 μm. (j-l) identification of PARP-2 in the top section of *V*. *faba* roots by the method of *tissue printing*, negative images. In the top left corner of each negative image, there is a miniature of the same fragment of nitrocellulose membrane in color, i.e. stained in the reaction of NBT/BCIP (j'-l'). (j-j') control, (k-k') HU, 32 h, (l-l') HU for 24 h and co-incubation HU/CF. *Scale bars* in j'-l' and j-l are 10 mm. (B) Identification of proteins H2AXS139Ph and PARP-2 by the method of Western blot. (a-a') expression levels of the H2AXS139Ph by Western blot analysis. Data shown are the representatives of three independent experiments. The relative levels of H2AXS139Ph after normalization for actin, as determined by densitometry analysis of the bands, are shown in the histogram (a'; the pixel values [*pv*; 1–255] categorized according to densitometry analysis of the band intensities and expressed in arbitrary units [a.u.]). *Columns*, mean from three independent experiments; *bars*, SD. * p ≤ 0.001 (Control/HU, Mann-Whitney *U* test); ▲ p ≤ 0.01 (Control/PCC, Mann-Whitney *U* test). (b-b') expression levels of the PARP-2 by Western blot analysis. Data shown are representative of three independent experiments. The relative levels of PARP-2 after normalization for actin, as determined by densitometry analysis of the bands, are shown in the histogram (b'; the pixel values [*pv*; 1–255] categorized according to densitometry analysis of the band intensities and expressed in arbitrary units [a.u.]). *Columns*, mean from three independent experiments; *bars*, SD. ▲ p ≤ 0.01 (Control/HU, Mann-Whitney *U* test); * p ≤ 0.001 (Control/PCC, Mann-Whitney *U* test).

**Fig 2:**
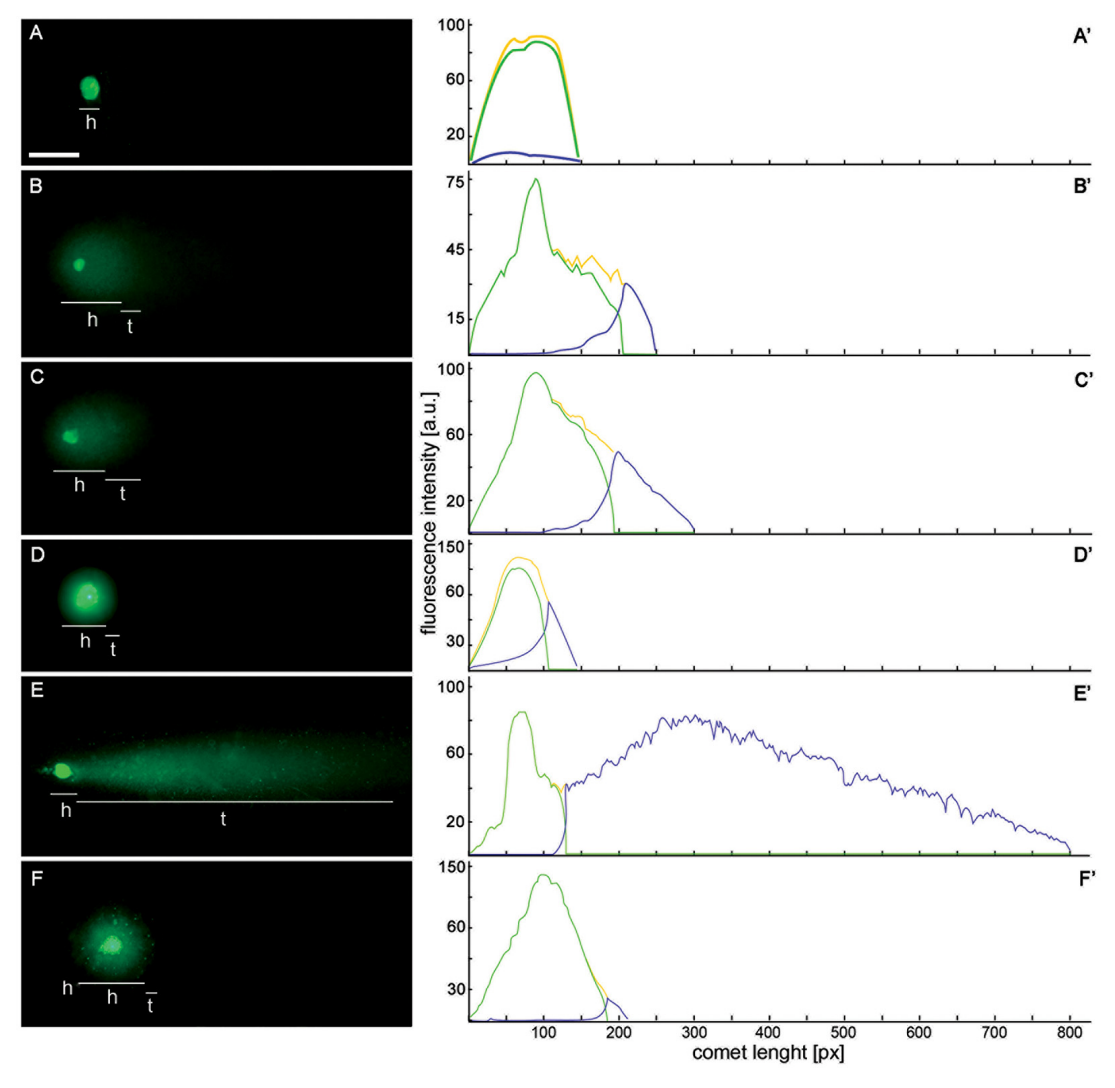
Single strand breaks (SSBs) and double strand breaks (DSBs) were assessed with alkaline (pH ≥ 13) and neutral (pH = 10) variants of comet assay, respectively. **SSBs were generated during replication stress (i.e. after 2.5 mM hydroxyurea [HU] treatment) and DSBs were connected with the induction of premature chromosome condensation (PCC) due to co-treatment with HU and caffeine (CF).** (A-F) fluorescence images of nuclei from individual protoplast of *Vicia faba*, stained with YOYO-1 after electrophoresis (comet assay); *h*, comet head; *t*, comet tail. (A-C) alkaline variant of comet assay dedicated to detection of SSBs; (D-F) neutral variant of comet assay assigned to DSBs. *Scale bar* in 'A' = 5 μm is applied to all figures presented. (A'-F') the intensity of DNA fluorescence after staining with YOYO-1 of the nuclei shown in (A-F). (A, D) nuclei of a protoplasts isolated from the untreated (control) *V*. *faba* roots. (B, E) nuclei of protoplasts isolated from the roots of *V*. *faba* treated with HU for 32 h. (C, F) nuclei of protoplasts isolated from the roots of *V*. *faba* treated with HU for 24 h and next co-treated with HU/CF for 8 h.

Previously we described that prolonged HU treatment led to rapid phosphorylation of histone H2A variant H2AX on S139 resulting in the formation of phospho-H2AXS139 foci along megabase chromatin domains near the sites of DSBs [37]. We also showed that the breakage of restrictive interactions of intra-S-phase checkpoints during PCC induction resulted in the accumulation of SSBs (co-locatization experiments using anti-ssDNA and anti-H2AXS139ph antibodies; [38]). Here, through quantitative immunocytochemical, tissue printing-related and biochemical analyses, we finally proved that both replication stress and PCC induction resulted in DNA damage (Fig 1A and 1B) and HU induced DSBs while HU/CF SSBs (Fig 1 and Fig 2).

Immunocytochemical identification of the phosphorylated form of H2AX histone was done with rabbit polyclonal antibodies (α-H2AXS139ph) directed against a synthetic peptide (CKATQA[pS]QEY) corresponding with a fragment of human H2AX histone (amino acids 134–142). According to our previous results [32–33] a small number of untreated cells emitted weak phospho-H2AXS139 fluorescence (2.1% ± 0.5) from areas distributed randomly within the cell nucleus (Fig 1Aa). H2AXS139 labeling index was significantly elevated in the HU-treated cells (61.9 ± 2.4; Fig 1Ab). In this series, apart from foci dispersed over the whole nucleus area, a new category of foci appeared, localized at the border of the nucleolus and nucleoplasm, probably connected with labeling of the perinucleolar heterochromatin fraction (Fig 1Ab). The addition of caffeine (HU→HU/CF), an inhibitor of superior kinases (of ATR/ATM type) that phosphorylates H2AX histones at serine 139, resulted in more than a 5-fold decrease in the quantity of specifically labeled nuclei, and additionally led to a decrease in the number of foci per nucleus from 16.9 ± 1.5 after HU to 7.5 ± 0.5 after HU+CF (Fig 1Ab and 1Ac). In the control series, the average number of foci per nucleus was 4 ± 1.0, while in the negative control (not incubated with primary antibodies) no cells were labeled (Fig 1Aa and 1Aa'). Immunocytochemical observations were confirmed by biochemical analysis (Fig 1Ad, Fig 1Ad', Fig 1Ae, Fig 1Ae', Fig 1Af, Fig 1Af', Fig 1Ba and 1Ba'). The *tissue printing* technique revealed strong phosphorylation of S139 in meristematic zones and a slightly weaker signal in supra-meristematic zones of roots treated with HU (Fig 1Ae and 1Ae'). In the roots co-treated with HU/CF, the positive reaction was limited to the meristem zone, while in the higher zones of the roots, strong signals were observed in the form of streaks, probably corresponding to the order of cells in the boundary zone between the primary cortex and the central cylinder (Fig 1Af and 1Af'). The results of the SDS-NuPAGE/Western blot analysis of the total extract from *V*. *faba* root tip cells revealed one band close to 16 kDa (Fig 1Ba)**,** as well as a strong increase (over 10-fold) in the amount of H2AXS139ph after HU-treatment, and an increase (over 4-fold) in the quantity of H2AXS139ph after co-treatment with HU/CF, in comparison with the control band on the same blot (Fig 1Ba and 1Ba'). The indicators point to the statistical significance of the results obtained (Mann-Whitney *U* test, p ≤ 0.001: * Control/HU; Mann-Whitney *U* test, p ≤ 0.01: ▲ Control/PCC i.e. HU/CF co-treatment in HU-synchronized cells).

Our previous results showed that labeling cell nuclei using antibodies recognizing *PARP2* gene product, i.e. poly(ADP-ribose) polymerase 2 (PARP-2), was an equally sensitive test detecting SSBs within DNA molecules [38]. Immunocytochemical analysis showed a low constitutive level of PARP-2 protein in the control cells (1.5% ± 0.3), an over 14-fold increase in PARP-2 protein after treatment with HU (21.3% ± 1.9) and specific labeling of almost half of the cells forming the root meristem (46.2% ± 2.8) in the series in which PCC was induced with CF (Fig 1Ag, Fig 1Ag', Fig 1Ah and Fig 1Ai). A 24-h incubation in 2.5 mM HU contributed to the formation of numerous fine specific PARP-2 foci, localized first of all in the perinucleolar region, as well as on the area of the entire nucleus and–in a characteristic way–on the periphery of cell nuclei in the region connected with the nuclear envelope (Fig 1Ah). The incubation in HU/CF, apart from an increase in the number of labeled cells, resulted in a considerable increase in the size of PARP-2 positive foci, as well as in disappearance of labeling in the perinucleolar heterochromatin and strong labeling of the boundary area of the nucleoplasm (i.e. those areas of the nucleus that adhered to the nuclear envelope; Fig 1A). In turn, the results obtained by the *tissue printing* method were not unequivocal, since strong labeling was observed in the case of root imprints derived from all experimental series (including the control); at the same time, a slightly more intensive labeling exactly of the meristematic zone was observed after the treatment with HU and after the PCC induction (Fig 1Aj, Fig 1Aj', Fig 1Ak, Fig 1Ak', Fig 1Al and Fig 1Al'). The results of the Western blot analysis of the total extract from *V*. *faba* root tip cells revealed only one band close to 66 kDa (Fig 1Bb) as well as showed a 5-fold increase in the amount of PARP-2 after HU-treatment and an over 11-fold increase in the quantity of PARP-2 after the HU/CF co-treatment, compared with the control band on the same blot (Fig 1Bb and 1Bb'). The indicators point to the following statistical significance: Mann-Whitney *U* test, p ≤ 0.01: ▲ Control/HU; Mann-Whitney *U* test, p ≤ 0.001: * Control/PCC i.e. HU/CF co-treatment in HU-synchronized cells. The performed analyses clearly show that both the formation of H2AXS139ph and PARP-2 foci are sensitive tests revealing the presence of structural damage to the genome (Fig 1A and 1B).

The type of DNA fragmentation can be also distinguished in *comet assay* [28,39]. Regardless of the method (alkaline or neutral variant), intact DNA remains in the head of the comet and DNA from regions with strand breaks appears in the tail. The amount of DNA in the tail is proportional to the number of DNA breaks. Fig 2A, Fig 2A', Fig 2B, Fig 2B', Fig 2C and 2C' present the alkaline variant of comet assay whereas Fig 2D, Fig 2D', Fig 2E, Fig 2E', Fig 2F and 2F' the neutral one. In the *V*. *faba* cells exposed for 24 h to HU, the percentage of DNA in the comet tails showed a higher level of DNA migration in both alkaline and neutral pH conditions. In the neutral variant, changes observed were as follow: 62% ± 1.1 of DNA in the comet tail for HU-treated cells, (Fig 2E and 2E'), control (2.9% ± 0.6; Fig 2D and 2D'); whereas in the alkaline variant: 34% ± 0.8 for HU-treated cells, Fig 2B and 2B', and for control 0.9% ± 0.3, Figs 2A and 1A'. During the PCC induction the results we observed were reversed, i.e. a higher level of DNA in the tail of comets in the alkaline version of the *comet assay*, compared to the natural version: 41% ± 2.3 and 20.6% ± 0.9, respectively. The results presented in Fig 2B', Fig 2C' and Fig 2E' are statistically significant (p < 0.001 compared to the control [presented in Fig 2A' and Fig 2D', respectively]; ANOVA and Tukey's *a posteriori* test).

### AL-PCD is a secondary result of CF-induced PCC in HU-synchronized *V*. *faba* roots

DNA cleavage is a PCD marker. Nucleases cleave DNA between nucleosomes which results in 180 bp fragments [3]. The effect of this process can be visualized on electrophoresis gel [3,8,28] indicating apoptotic cells (ladder pattern) and showing immediate DNA degradation during necrosis (smear), while DNA in living cells is not fragmented [3,28].

To determine whether the PCC induction is connected with PCD-type DNA degradation, we separated the isolated samples of DNA on agarose gel and visualized under UV light. Electrophoresis showed a large scale DNA fragmentation only in the HU/CF co-treated series (S2 Fig, lane 3, arrows). Laddering was not detected in the HU-incubated series nor in the control (S2 Fig, lane 2 and lane 1, respectively). Typical internucleosomal DNA fragmentation was undetectable.

**Fig 3:**
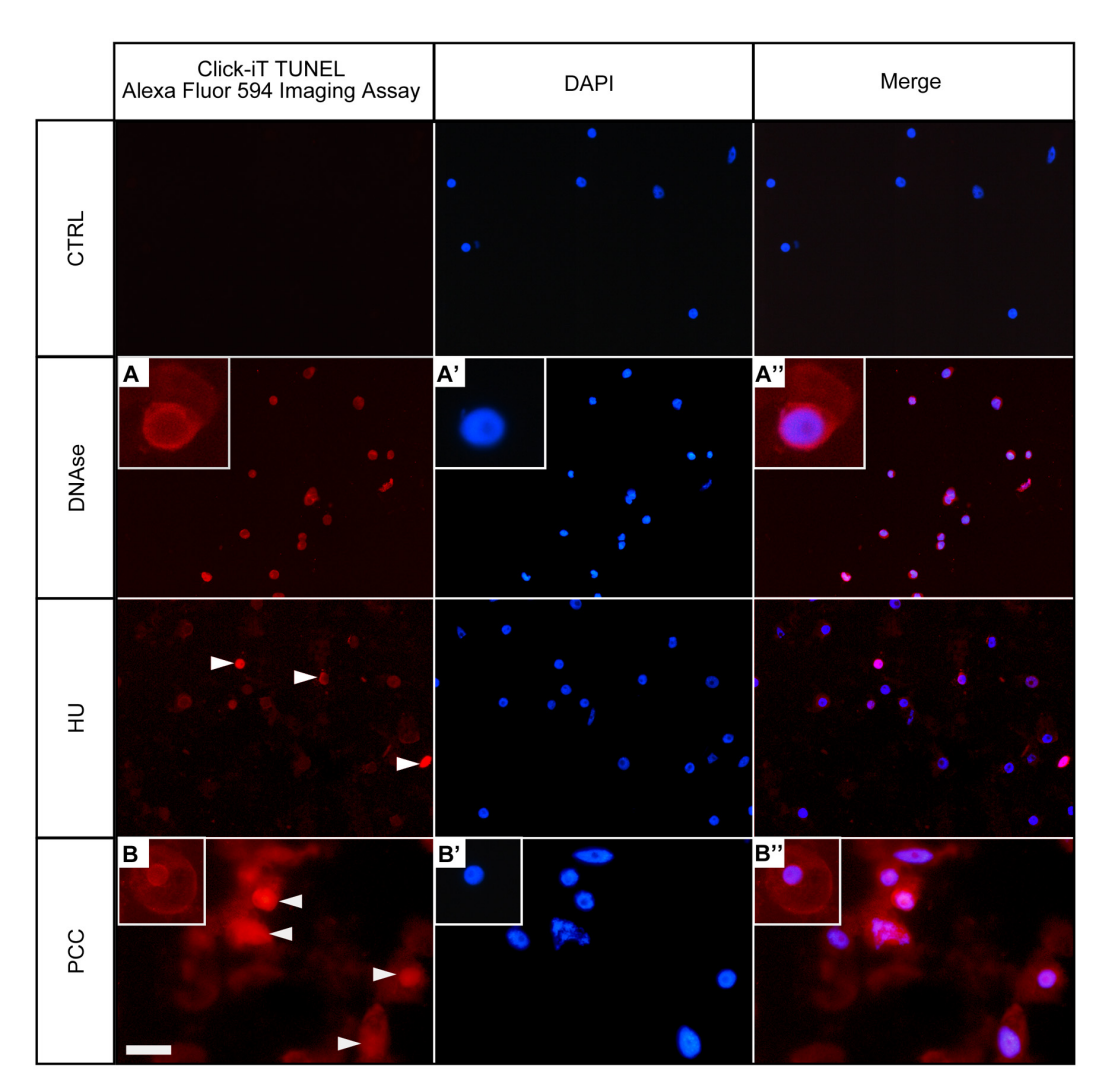
Terminal deoxynucleotidyl-dUTP nick end labeling (TUNEL) assay in Click-iT technology in the untreated control (negative), DNase-treated control (positive), HU-treated and HU/CF-co-treated (i.e. PCC-induced) *Vicia faba* root meristem cells. *Left panel*—DNA fragmentation in *V*. *faba* cells detected by TUNEL reaction and visualized by AlexaFluor 594. *Central panel*—DAPI stained nuclei. *Right panel*—Merged images (AlexaFluor 594 + DAPI). Positively stained nuclei appear in the DNase-treated cells (e.g. A-A'') and in the HU/CF co-treated cells (e.g. B-B''). Positively stained nuclei in the HU-treated series are indicated by arrowheads. Non-reacting nuclei can be seen in the negative control sections (as indicated in the *highest panel* described as 'CTRL'). *Scale bar* = 20 μm.

Terminal deoxynucloetidyl transferase-mediated dUTP nick and labeling (TUNEL) assay, which positively stains apoptotic nuclei, can be also used to determinate the type of DNA fragmentation. TUNEL assay allows determination of the presence of free 3’-OH ends in chromatin [40]. The TUNEL assay was used here to detect 3'-OH termini in nuclear DNA (Fig 3). For DNase I-treated series (positive control), all cells were TUNEL-positive (Fig 3A, Fig 3A' and Fig 3A''). In the control series (32-h incubation in water) no TUNEL reaction was evident as no AlexaFluor 594-related red fluorescence was observed (Fig 3, the top panel). In the HU-treated series, we observed a TUNEL-positive sign in a small part of meristematic cells only (< 11% ± 1.3; Fig 3). In contrast, the cell population treated with the mixture of HU/CF exhibited up to 48% ± 2.4 of TUNEL-positive nuclei (Fig 3B, Fig 3B' and Fig 3B'', the bottom panel, TUNEL-positive indicated by arrows). Statistical results analyzed by Student *t*-test indicate that differences in the percentage of TUNEL-positive cells between the control and HU-treated cells, as well as between the control and PCC-induced cells (i.e. HU/CF co-treated) are significant, p ≤ 0.01.

**Fig 4:**
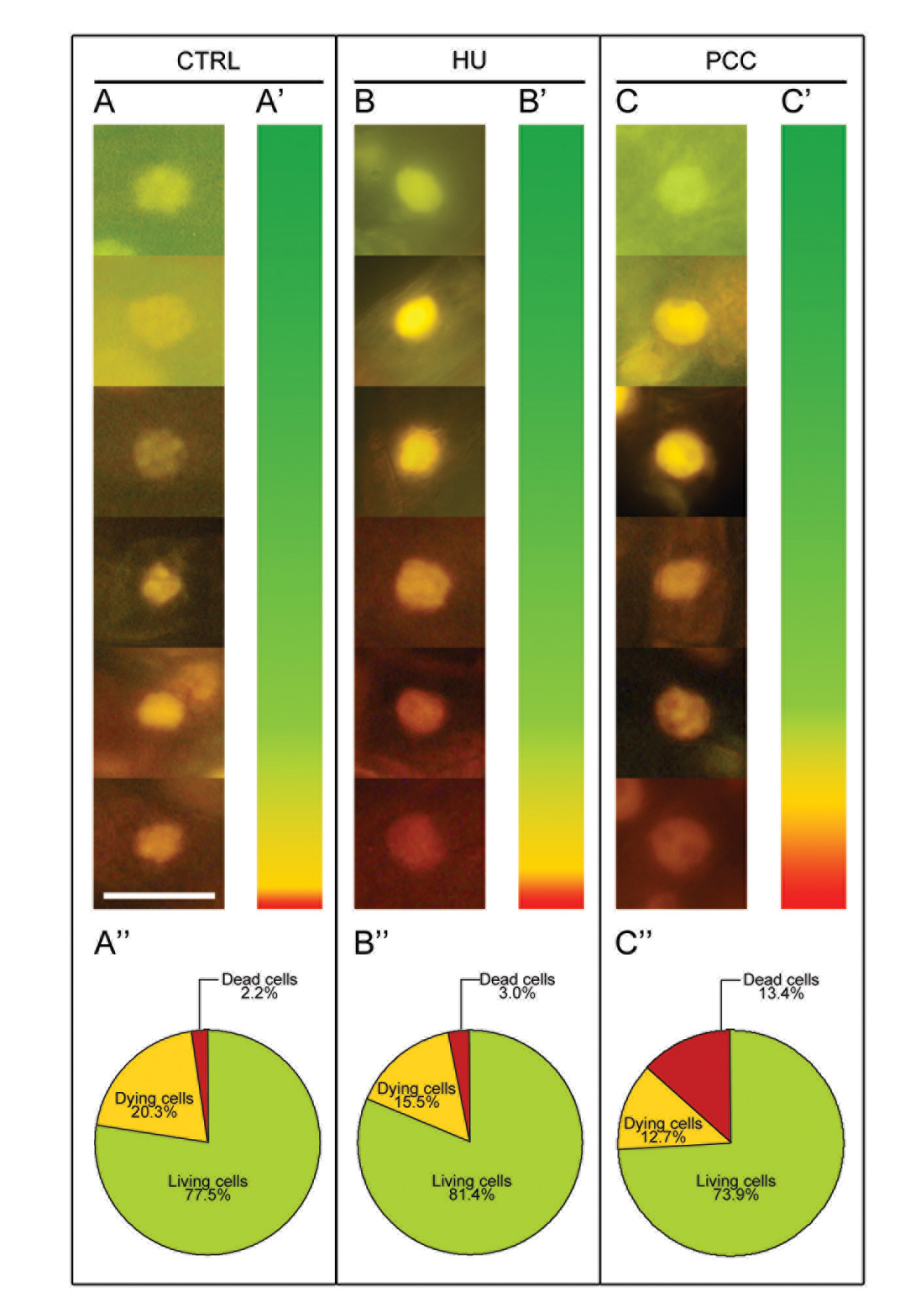
Double *in vivo* staining with acridine orange (AO) and ethidium bromide (EB) as a useful tool for detecting and quantifying the state of dead, dying and living cells in root meristems of *Vicia faba*. (A-A'') control. (B-B'') hydroxyurea-induced replication stress. (C-C'') caffeine-induced premature chromosome condensation (PCC). (A,B,C) fluorescence micrographs of nuclei in living (green), dying (range: yellow-to-orange), and dead (red) cells. (A',B',C') diagrams presenting the color spectrum resulting from measurements of the fluorescence intensity of nuclear chromatin stained with AO/EB, in order to determine the degree of damage in the nuclei of stressed roots of *V*. *faba*. (A'',B'',C'') circle diagrams presenting the percentage of living (green), dying (range: yellow-to-orange) and dead cells (red). The data shown in the pie charts in A'',B'',C'' indicate that the correlations were significant with reference to the number of dead cells for all experimental series reported herein: an association was found between the control and HU (p ≤ 0.05, Mann-Whitney *U* test), between the control and PCC (p ≤ 0.01, Mann-Whitney *U* test), and between the HU-treated and PCC-induced cells (i.e. HU/CF co-treated; p ≤ 0.01, Mann-Whitney *U* test). *Scale bar* in (A) = 20 μm is also applied to the figures presented in the pictures (B) and (C).

**Fig 5:**
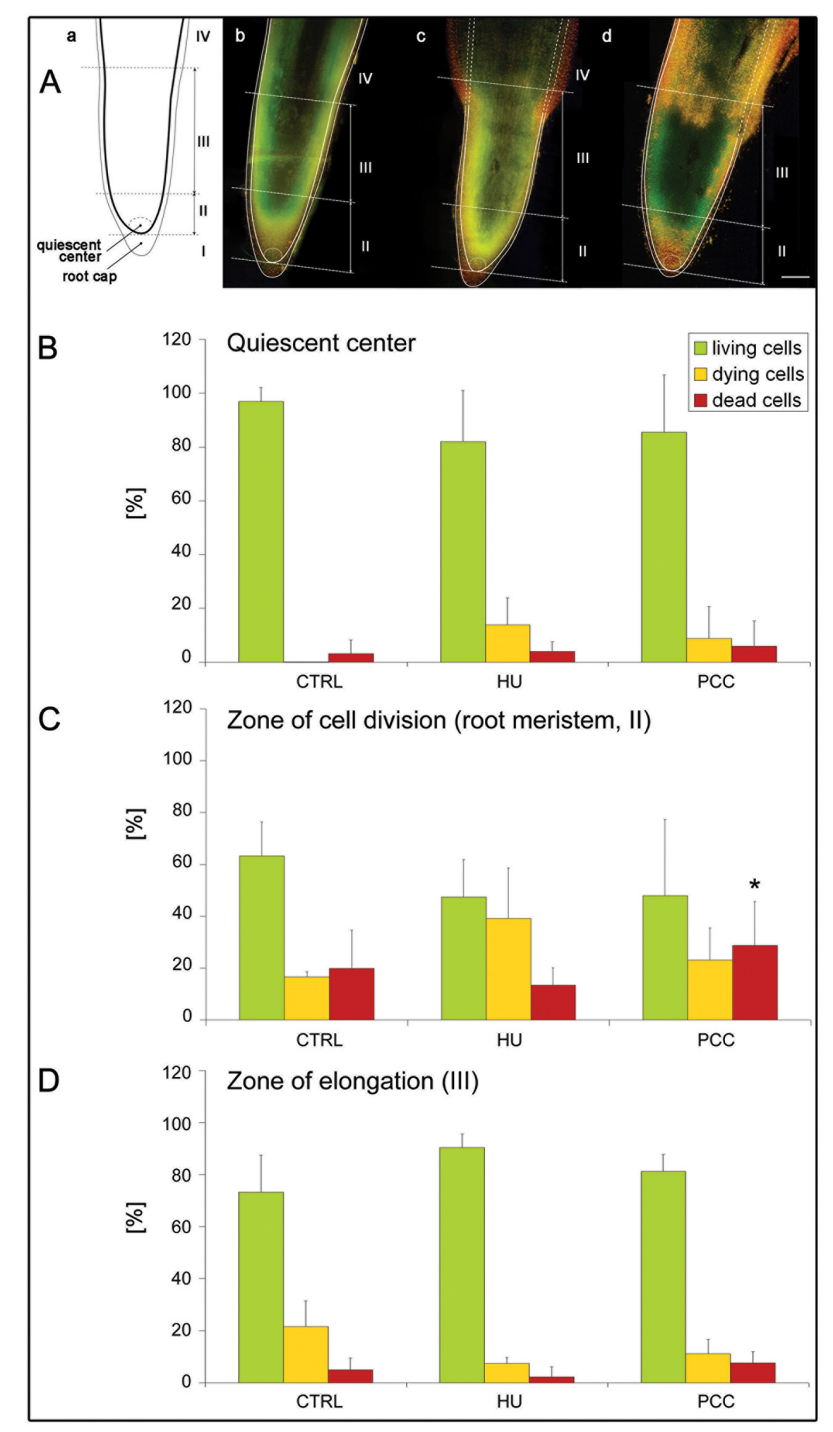
Acridine orange (AO) and ethidium bromide (EB) staining of living, dying and dead cells in *Vicia faba* root meristem cells in relation to (A) the localization in particular zones, and (B-D) the surface area occupied by the cells in particular zones. (A) Fluorescence picture of AO/EB stained *V*. *faba* roots *in planta*. (a) schematic figure presenting the outline of the roots of the control series, with marked (I) root cap, (II) zone of cell division i.e. root meristem, (III) zone of elongation, and the quiescent center. (b) the control roots, (c) the roots treated with 2.5 mM hydroxyurea (HU) for 32 h, (d) the roots treated with 2.5 mM HU for 24 h and then co-treated with 2.5 mM HU and 5 mM caffeine (CF). *Scale bar* = 1 mm. The schematic outline of the root from scheme (a) was placed over a root from the control series (b) on which the root outline from figure 'a' and figure 'b' are precisely overlapped, (c) a root from the series, in which seedlings were subjected to replication stress and (c) a root that was induced to premature chromosome condensation (PCC). (c-d) The continuous line marks those root fragments that in terms of size and shape were the same as the analogous areas in the roots of the control series (a-b *versus* c-d), while the broken line (in figures [c] and [d]) marks the root areas that indicated the appearance of aerenchymatic-like spaces that had formed in the roots that had been subjected to treatment with HU (c) or co-treated with the mixture of HU/CF (d). In places indicated by broken lines, roots of the series (c) and (d) were distinctly wider than the control (b). (B-D) quantitative presentation of surface area (%) occupied by the green, yellow-orange and red colors (that correspond to the populations of living, dying and dead cells, respectively) in the particular zones of *V*. *faba* roots. (B) quiescent center, (C) zone of cell division, i.e. root meristem, marked also as zone II, and (D) zone of elongation, marked as zone III.

Double staining with acridine orange (AO) and ethidium bromide (EB) is another method used to specify the type of cell death, as well as being a useful tool in detecting and quantifying the states living, dying and dead (Fig 4, Fig 5 and S3 Fig). Fluorescence microscopy revealed images indicating the occurrence of living cells (fluoresce green), dying cells (green-yellow, yellow, yellow-orange and bright orange) and dead cells (dark orange and bright red) in the control (Fig 4A), after both HU treatment (Fig 4B) and after PCC induction (Fig 4C). The diagram presenting the color spectrum resulting from the quantitative measurements of fluorescence intensity of nuclear chromatin stained with AO/EB was made in order to determine the degree of DNA damage in the control nuclei (Fig 4A') as well as in the nuclei derived from stressed roots of *V*. *faba* (Fig 4B and 4C'). The highest number of dead cells (13.4%) was observed in HU/CF treated material (Fig 4C''). Thus, it was shown that the number of dead cells after PCC induction was over 6-fold higher compared to the control and 4.5-fold higher, compared to the HU series (Fig 4A'', 4B'' and 4C''). The fractions of living, dying and dead cells in the control, compared with the HU series were similar; we only observed an increase in the number of dead cells in the latter (1.5-fold; Fig 4A'' and 4B''). Further analysis consisted in comparing the numbers of living cells or being at various cell death stages (early-to-late) in particular zones of *V*. *faba* roots [from 'quiescent center' through the zones of cell division, i.e. root meristem (zone marked as II in Fig 5A) up to the zone of elongation (marked as III in Fig 5A)]. Our results suggest that the increase in the number of dead cells in the meristematic zone of *V*. *faba* roots after HU/CF treatment was statistically significant (p < 0.01) in relation to both the control and HU-treated series (Fig 5C). In the meristematic zone (II) the relatively high percentage (Fig 5C) populations of dying (Fig 5C, *yellow-colored columns*) and dead cells (i.e. 19.9%, 13.4%, and 28.8% in the control, HU-treated, and PCC-induced series, respectively) resulted from some imperfection in the method of intravital AO/EB staining, and from the fact it is not possible to omit from the calculation the population of rhizodermis cells in which the occurrence of the PCD phenomenon is typical (comp. Fig 5Aa, Fig 5Ab, Fig 5Ac, Fig 5Ad and Fig 5C; this could be the reason that no significant differences were observed herein). In the other zones of *V*. *faba* roots induced to PCC, the number of dead cells was about 1.5-fold higher than in the control and almost 2-fold higher than in the HU-treated material (Fig 5A and 5B). In turn, in the elongation zone (III, over-meristematic, Fig 5A), the highest index of dead cells was observed under the influence of HU/CF (7.7%, i.e. 1.5-fold and 3.4-fold higher than in the control and HU-series, respectively; Fig 5D). However, it seems more interesting to compare the numbers of dead cells in the meristematic zone than in the elongation zone; in the latter it was lower, where in the control, the HU and the HU/CF series, decreases of 4, 6 and 3.7 times were observed, respectively (comp. Fig 5C and 5D). S3 Fig shows longitudinal intersections of the three most representative roots stained with the intravital AO/EB method, as well as shows the proportions of living (green), dying (range: yellow-to-orange) and dead (red) cells, and their distribution in the meristem, supra-meristematic zone and in the rhizodermis. In all zones (particularly in the area of a root cap) and in all experimental series we observed red fluorescence indicating PCD processes eliminating cells, particularly from the rhizodermis, present in the external layers of the root (S3 Fig). The arrows on S3b, S3b' and S3b'' Figs show a distinct widening of the HU-treated roots forming an easily visible protuberance in which the accumulation of dead cells can be observed. These protruberances may result from the appearance of aerenchymatic-like spaces in the root cortex cells of *V*. *faba* (comp. [8]).

### Vacuolar/autolytic (V/A) AL-PCD, following CF-induced PCC in HU-synchronized *V*. *faba* roots

**Fig 6:**
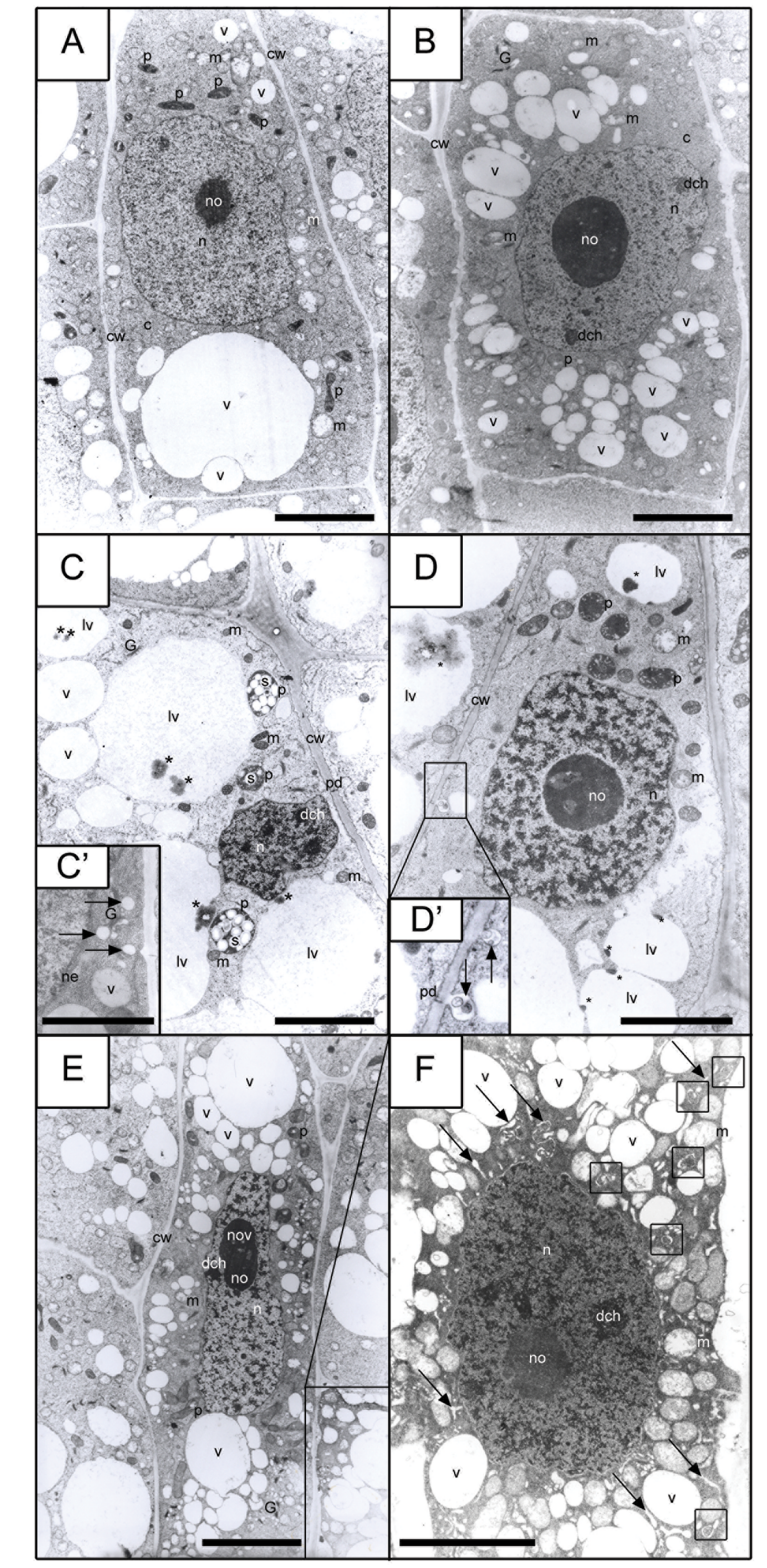
Electron micrographs of *Vicia faba* root meristem cells. (A) control (32-h incubation in water); (B) hydroxyurea-treated (32-h); (C-C', D-D', E-F) hydroxyurea (HU) synchronized for 24 h and then HU/CF co-treated (for a successive 8 h; total incubation time: 32 h). The arrows in picture (C') point out vesicles of the Golgi apparatus. The arrows in picture (D') indicate lytic vacuoles localized in the vicinity of plasmodesmata. The square in the bottom right corner of picture (E) contains an enlarged image of the cell from picture (F). Asterisk (*), the visible light in the vacuoles presented in pictures (C and D) indicates the places of accumulation of deposits in the vacuoles, showing that these vacuoles function as lytic vacuoles. All the photos presented in figures (C-F) are derived from the series in which PCC was induced. However figure (C) shows the morphology of the root cuticle cells, from which the plastids seen in the picture (precisely amyloplasts, marked as '*p*') are filled with statolith starch grains (marked as '*s*'). Successive figure (D) presents the appearance of a typical *V*. *faba* meristematic cell, whose morphology (apart from the deposits seen in the lytic vacuoles and indicated by the asterisk) does not significantly differ from the morphology of the control cells (comp. A and D). Two further pictures (E and F) illustrate the morphology of meristematic cells that entered the path of apoptosis-like programmed cell death (AL-PCD), while picture (E) shows premature vacuolization stadium, and picture (F) demonstrates: (1) extensive vacuolization within the whole meristematic cell space, (2) the presence of swollen ER compartments (indicated by arrows), and (3) the existence of autophagosome-like structures, created from ER (the structures inside the squares). *a-l* autophasome-like structure, *c* cytoplasm, *cw* cell wall, *dch* dense chromatin, *ER* endoplasmic reticulum, *G* Golgi structure, *lv* lytic vacuole, *m* mitochondrion, *n* nucleus, *ne* nuclear envelope, *no* nucleolus, *nov* nucleolus vacuole, *p* plastid, *pd* plasmodesmata, *s* starch, *v* vacuole. *Scale bar* = 5 μm.

**Fig 7:**
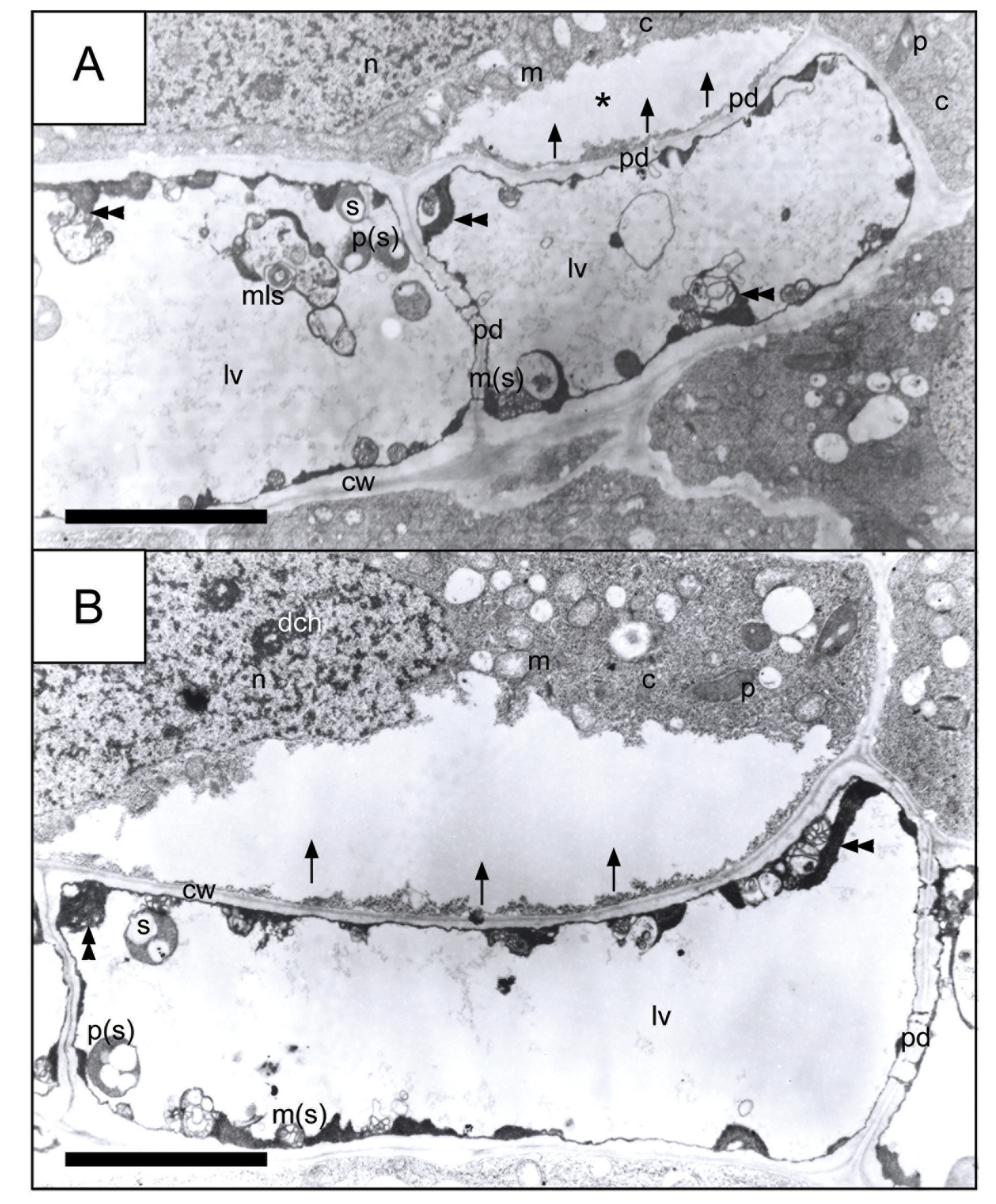
Spreading of (presumably) lytic enzymes from the interior of almost totally degenerated dead cells to adjacent cells through plasmodesmata. (A-B) The direction of enzyme propagation is indicated with arrows. Double arrow-heads indicate cytoplasm residues pushed towards plasmalemma with suspended organelles already partially digested. The asterisk in picture (A) indicates electron lucent areas, newly formed inside a regular meristem cell (i.e. a cell that does not show changes in other regions, other than those marked with an asterisk where the wave of lytic enzymes arrived). Picture (B) Gradual, sequential propagation of a cleared up region appearing, presumably, due to the action of lytic enzymes in a cell neighboring a dead cell due to AL-PCD, towards further areas of the adjacent cell. *c* cytoplasm, *cw* cell wall, *dch* dense chromatin, *lv* lytic vacuole, *m* mitochondrion, *mls* multilamellar structure; *n* nucleus, *p* plastid, *pd* plasmodesmata, *s* starch; *p(s)* swollen plastids; *m(s)* swollen mitochondium. *Scale bar* = 5 μm.

The aberrant course of prematurely induced mitotic division as a rule leads to cell apoptosis, PCD (or AL-PCD in plant cells). In order to establish a possible cause-and-effect relationship between the induction of PCC and of AL-PCD, and to determine the type of AL-PCD, precise ultrastructural investigations were performed of the meristematic zone in *V. faba* roots. The results presented in Figs 6 and 7 and S4–S7 Figs show that PCC induction increased the number of root cells with AL-PCD symptoms, and that the electron microscope images distinctly indicate the occurrence of a vacuolar/autolytic type of AL-PCD [(V/A) AL-PCD].

The AL-PCD process is connected with several metabolic-biochemical changes in the cell. They can concern either the nuclear compartment or the extranuclear regions (in the latter case these can be connected with changes observed in the cytoplasm or organelles present in it). Early AL-PCD events were found to be connected with changes occurring in the cytoplasm [e.g. progressing vacuolization and formation of greater and greater lytic vacuoles (see Fig 6B, Fig 6C, Fig 6D, Fig 6E and 6F in comparison with Fig 6A)]. It has also been shown that the beginning of changes concerning the nuclear compartment took place only when the changes observed in the cytoplasm were considerably advanced (comp. Fig 6F with Fig 6D). Unexpectedly, no changes were observed in the structure of mitochondria (S4C Fig).

One of the first symptoms of AL-PCD concerning the nucleus was a considerable increase in the condensation degree of chromatin fibrils (the first stadium of this process is shown in Fig 6F, while an advanced stadium is presented in S5A and S5C Fig). The occurrence of the V/A-type of AL-PCD induced during the co-treatment with HU/CF was indicated on the basis of the following symptoms: (i) extensive vacuolization within the whole cell space (early stages of vacuolization are presented in Fig 6E, and late stages in Fig 6F), (ii) the presence of deposits within the lytic vacuoles (Fig 6C and 6D), and (iii) the existence of autophagosome-like structures created from, among other things, the swollen ER (Fig 6F). AL-PCD symptoms were not observed in either the control series or the HU series (S4A and S4B Fig). The majority of cells induced to enter PCC also showed no AL-PCD symptoms (apart from insignificant changes in their morphology and e.g. the formation of vacuoles with a lytic character; S4C Fig). However 5.3% ± 1.1 of cells subjected to PCC entered the (V/A) AL-PCD pathway. The qualification of particular cells to those in which (V/A) AL-PCD symptoms were detected took place when changes indicating the occurrence of AL-PCD concerned the nuclear compartment (S5A, S5B and S5C Fig). We used this classification in this paper, which is consistent with Dominguez and Cejudo [41], who considered the degradation of cellular nucleus to be the symptom of the final and irreversible stage of PCD (although the final degradation of nucleus was caused by metabolic changes, for example those occurring in the cytoplasm in cells undergoing PCD).

The electron microscopy observations of cells induced to PCC and then entering the AL-PCD pathway showed that the most visible changes took place in the nucleus. In the *V*. *faba* nuclei the increasing transparency of decondensed nucleoplasm was the basic morphological indicator of the successive stages of AL-PCD. In addition, it served as a convenient background against which it was easy to distinguish the extremely condensed fibers of condensed chromatin. These strongly condensed areas of chromatin were often adjacent to the nuclear envelope (S5A and S5C Fig). The other characteristic features indicating the occurrence of AL-PCD include, among others: (1) shrinkage of the protoplast (S5B Fig); (2) formation of sections of a multi-layer nuclear envelope (S5A and S5C Fig); (3) formation of multi-membrane structures either in the region of plasmalemma or nuclear envelope (S5A, S5B, S6B and S5C Figs); (4) degradation of organelles inside lytic vacuoles (S5A, S6B, S6C, S7A and S7B Figs); and (5) formation of autophagosome-like structures (S5C Fig). Additionally, the triggering of (V/A) AL-PCD was accompanied by the appearance of specific structures inside the cell: (1) either showing indistinct/unclear morphology (cloudy morphology; S6A, S6B and S6C Fig); or (2) having a clear myelin character (S6B Fig). It was also shown in this work that the 'signal transmission' (from one cell to another cell) proceeded, among other things, through plasmodesmata (Fig 7, comp. Fig 6D and 6D'), i.e. cytoplasmic channels selectively displacing metabolites and signal molecules present inside lytic vacuoles (Fig 6D and 6D'). The cytoplasm of the cells showing symptoms of (V/A) AL-PCD was relatively bright, as caused by the reduction in the number of ribosomes (S6B, S7A and S7B Figs). Plastids, mitochondria and other organelles were gradually pushed towards the cell walls (S5B, S7A and S7B Figs). Compact Golgi structures accompanied by quite large vesicles filled with an electron-transparent material (Fig 6C') were easily distinguishable (Fig 6B and 6E).

Finally, fragmentation of the nuclei and their progressing marginalization were among the final stages of (V/A) AL-PCD proceeding in the meristematic cells of *V*. *faba* root (however, this stage was observed only when almost all the organelles in a given cell were subjected to degradation by -presumably—lytic enzymes). The description of the final stage of cell degradation should be as follows: when the cell interior is almost totally filled with a huge lytic vacuole and most organelles have been degraded (and those that have not been completely digested are pushed towards border cell areas, towards plasmalemma), organelles show strong changes in their morphology; changes that resemble swelling from the long-lasting influence of (presumably) lytic enzymes on the intercellular structures and preceding the moment of their final digestion (Fig 7A and 7B). Fig 7 also showed that a cell that had died as a result of (V/A) AL-PCD was still able to transmit a stream of lytic enzymes derived from its own lytic vacuole through the system of plasmodesmata into an adjacent cell (even when the morphology of the adjacent cell was normal).

**Fig 8:**
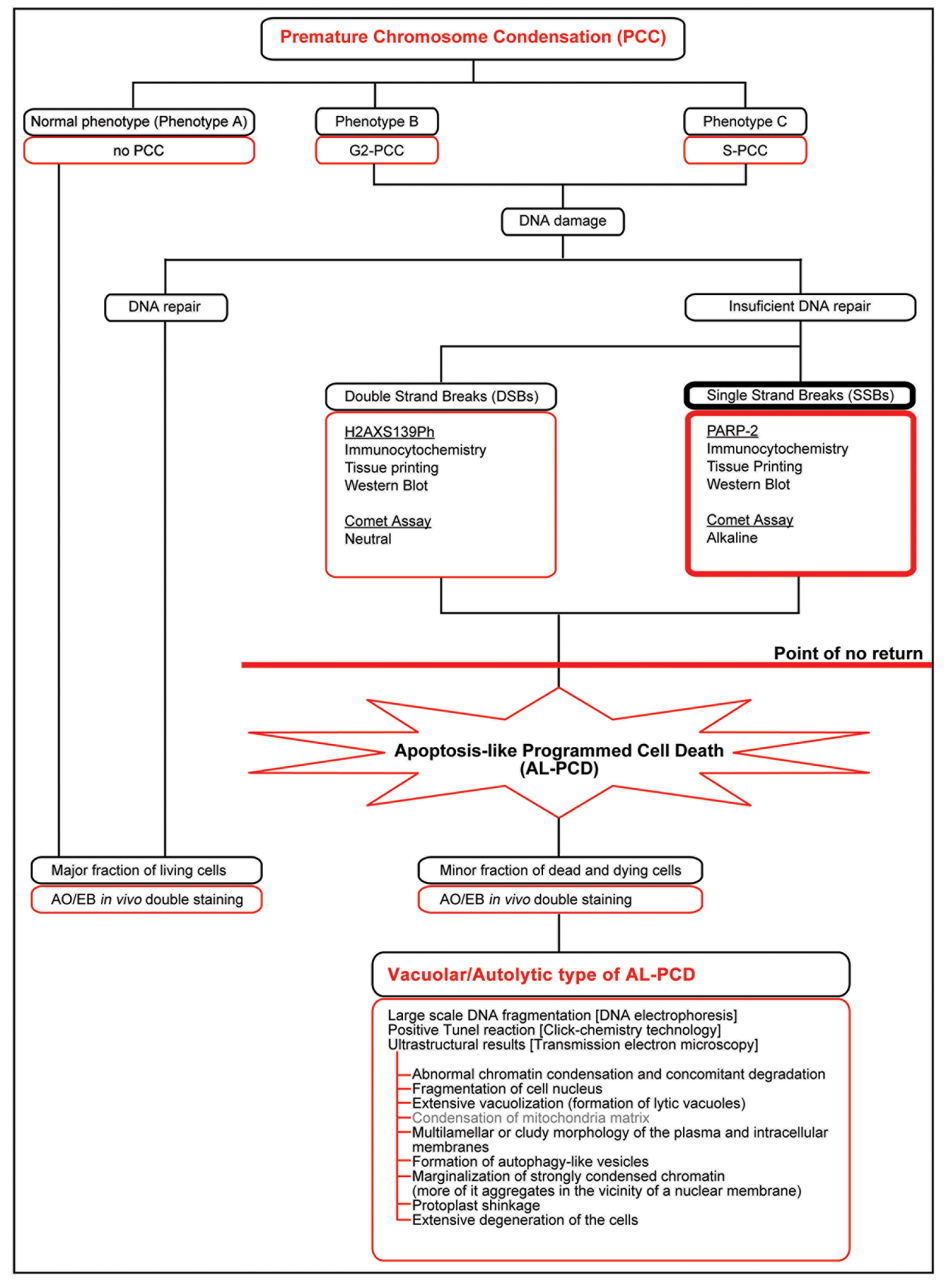
Scheme depicting experimental procedures used to reveal and to support the fact that the activation of vacuolar/autolytic type of plant-specific, apoptosis-like programmed cell death [(V/A) AL-PCD] is a secondary result of the caffeine-induced premature chromosome condensation (PCC) in hydroxyurea (HU) synchronized *Vicia faba* root meristem cells. The scheme shows that PCC leads to the formation of cells with different phenotypes (phenotype A–typical of cells not diverging from the morphology of regular cells; phenotype B–characteristic of cells with the morphology typical of PCC induction from G2 phase; and phenotype C–cells with strongly fragmented chromosomes, induced to PCC, probably in early or middle sub-periods of S phase). The scheme shows that the induction of PCC is associated with the generation of DNA damage (mainly single-strain breaks [SSB] but also to a lesser extent, double-strain breaks [DSBs]). The occurrence of various types of DNA damage during PCC induction has been proven following the results of many experimental procedures (immunocytochemistry, with the use of antibodies against specific marker proteins, *tissue printing* methods, Western blot and *comet* tests–both alkaline and neutral). In turn, double staining with acridine orange and ethidium bromide allowed us to distinguish *in planta*, the sub-fractions of living, dying and dead cells. Preliminary histochemical analyses (classic staining with Schiff’s reagent in Feulgen’s method) showed the presence of such changes in the structure of cellular nuclei that could indicate the occurrence of apoptosis-like programmed cell death (AL-PCD). On the other hand, more detailed analyses, i.e. DNA electrophoresis and TUNEL reaction, confirmed by ultrastructural tests (performed at a level of transmission electron microscopy) proved the existence of vacuolar/autolytic type of AL-PCD (V/A-type AL-PCD) in stressed *V*. *faba* roots.

The results of the investigation performed (summarized in Fig 8) allow us to put forward the thesis that the induction of (V/A) AL-PCD in the *V*. *faba* cells may, and even should, be perceived as a consequence of previously initiated PCC process and the DNA damage occurring during its course.

## Discussion

The major finding of this paper is that CF/HU-induced PCC triggered the AL-PCD pathway in the root meristem cells of *V*. *faba*. We categorized this phenomenon as (V/A) AL-PCD, i.e. vacuolar/autolytic type of plant-specific PCD, according to the nomenclature introduced by van Doorn in 2005 [42] and in successive works of the Nomenclature Committee on Cell Death (NCCD), also taking into consideration the systematization of knowledge about PCD-related terms [19–20].

Previous experiments revealed that PCC induced by 8 hours of incubation in a mixture of HU/CF was characterized by a strong differentiation of the morphological forms of chromosomes. Three different phenotypes could then be distinguished: A, B and C. 'Phenotype A' cells had morphology similar to that of normal mitotic cells (normal phenotype = phenotype A = lack of visible PCC symptoms; S1 Fig). 'Phenotype B' cells seemed not able to finish the post-replication repair processes in the G2 phase, i.e. just prior the onset of CF-induced PCC (phenotype B = G2-PCC; S1 Fig). 'Phenotype C' cells showed a considerably higher degree of chromatin fragmentation, and entered PCC despite unfinished DNA replication in the S phase (phenotype C = S-PCC; S1 Fig). The generation of PCC-related damage is connected either with PCC induction or PCC progression.

In the present work, the presence of DSBs was confirmed by a neutral version of comet assay, and the discovery of phosphorylation of histone H2AX on S139 (H2AXS139Ph; Fig 1). In turn, the presence of SSBs was confirmed by an alkaline version of comet assay and the presence of poly(ADP-ribose) polymerase-2 (PARP-2), i.e. a protein considered to be a marker of SSBs (Fig 1; comp. [38]).

Several methods have been developed to determine PCD occurrence and distinguish its type. Fluorescein diacetate (FDA) can be used to distinguish PCD from living cells and apoptosis or AL-PCD from necrotic death. Living cells show fluorescence of FDA, PCD do not show fluorescence but protoplasts become detached from cell walls and in necrosis neither fluorescence nor protoplast detachment is observed [3]. In contrast, the use of double staining with AO and EB showed that a considerable number of cells co-treated with HU and CF had survived and remained alive (Fig 4 and Fig 5); by activating mechanisms connected with DNA repair (Rybaczek, in preparation). Some of the cells previously subjected to PCC showed the features of (V/A) AL-PCD (5.3% ± 1.1) and were stained either red in AO/EB testing (dead cells; Fig 4, Fig 5 and S3 Fig) or yellow-orange (dying cells; Fig 4, Fig 5 and S3 Fig). In these cells, damage had overwhelmed the repair mechanisms. The method of intravital dual AO/EB staining was first used to assess the viability of animal cells [43] and was then adapted to the model of *V*. *faba* cells [8]. The principle of the method is that AO (staining DNA green) has the ability to penetrate into a nucleus regardless of the state of cell membranes. In contrast, EB (staining nuclei red) requires an increased permeability of the nuclear membrane. Classification of the particular color ranges corresponding to the individual stages of the type of cell death, is derived from the PCD induction model in hybrid tobacco cells treated with high levels of cytokinin BAP [44], as well as from the paper by Byczkowska et al. [8] describing the cell death phenomenon in *V*. *faba* root meristem cells treated with 1-aminocyclopropane-1-carboxylic acid (ACC).

In dead and dying cells, the 'point of no return', as described by van Doorn [42], was reached and/or exceeded, and consequently the pathways connected with the process of cell death were initiated (Fig 8). Similar results were achieved in naphtoquinones-treated tobacco BY-2 cells [45]. Furthermore, the ability of a secondary metabolite chalcone to induce PCD was demonstrated on *Arabidopsis thaliana* seedlings model [46]. The following signs of PCD were then revealed: mitochondrial condensation, disruption of organelles and chromatin condensation [46]. Additionally, as observed in mouse early embryonic ATR^-/-^ cells, apoptosis is caused by a loss of genomic integrity [47]. In this, genomic instability is induced by the accumulation of a high degree of chromosomal fragmentation caused by mitotic catastrophe (MC), i.e. 'premature entry into mitosis prior to the completion of the S phase and characterized by a high degree of chromosomal fragmentation' [48].

In this paper the onset of PCC was also associated with abundant chromosomal fragmentation (S1 Fig, Fig 1 and Fig 2), and in our opinion this kind of extensive PCC-related mitotic-type DNA fragmentation would likely contribute to the initiation of AL-PCD in *V*. *faba*. The eidetic definition of MC is that 'MC might not even constitute a *bona fide* cell death executioner mechanism, but rather an oncosuppressive pathway that precedes and is distinct from, yet operates through, cell death or senescence' [20,49–50]. It must be added that MC can also occur in the absence of apoptosis [51]. Despite this, the induction of apoptosis occurs most frequently during drug-induced PCC (comp. [16]), which suggests the co-existence of a similar signal transduction pathway toward apoptosis and PCC [11,15].

The occurrence of AL-PCD in *V*. *faba* root meristem cells induced to PCC then exposed to CF, was also confirmed by the positively stained nuclei in the TUNEL assay, as well as by the large-scale DNA fragmentation revealed in DNA electrophoresis. Oligonucleosome fragmentation of DNA, which increases with the level of chromatin condensation as well as chromatin disintegration (resulting from, among other things, the destruction of scaffold attachment factor A, SAF-A, by caspases [52]), is a characteristic feature of PCD in both plant and animal cells. Interestingly, under kinetin-induced PCD in the model of *V*. *faba* root meristem cells, DNA degradation was not observed as a typical internucleosomal process, but rather showed as 'a smear' indicating an exonucleolytic type of DNA degradation [53], despite the internucleosomal DNA degradation observed during PCD in other plants [41,54–55]. Following this, more detailed research by Kunikowska et al. [53] revealed digestion by acidic and basic nucleases of faba bean DNA in kinetin-treated (72 h) seedlings [56].

There are three major types of PCD depending on the stimulus and/or cells involved: (i) classical apoptosis (caspase-dependent mechanism); (ii) apoptotic-like PCD (AL-PCD; caspase-independent [5,41,57]), and (iii) necrosis-like PCD (caspase-independent). Each type of PCD leads to cell death, although with differing fragmentation of nuclear DNA. The 'classical apoptosis' pathway involves internucleosomal DNA fragmentation. For 'necrosis-like PCD' DNA fragmentation may not occur at all or occurs only as a 'secondary, lumpy-type DNA fragmentation'. In turn, the AL-PCD pathway (studied in this research) is distinguished by large-scale DNA fragmentation [58].

As PCD is an integral part of plant development, we attempted to categorize plant PCD in relation to its various morphological forms: (i) apoptosis; (ii) non-lysosomal PCD; including three more that can occur concurrently and can be followed by mega-autophagy, i.e.: (iii) micro-autophagy; (iv) macro-autophagy, and (v) mega-autophagy [59]. The type of cell death, and even the nature of the accompanying nuclear DNA fragmentation, appear to depend not only on the cell type (and species) but even on the experimental system used [8,30,40,53,56,60]. For this reason in this work (as in the vast majority of other studies) the DNA fragmentation during PCD evaluated by DNA electrophoresis needed to be confirmed by TUNEL reaction. Specific DNA degradation into oligonucleosomal fragments in tobacco BY-2 cells was shown as a result of cadmium treatment [60]; typical 'DNA laddering' during DNA electrophoresis was revealed during AL-PCD in *Pisum sativum* root tips exposed to sudden flooding [30] as well as during the aging of sunflower seeds [40]).

Detailed micrograph analyses based on TEM strongly supported the occurrence of the V/A-type of AL-PCD in *V*. *faba* seedlings following PCC induction. Characteristic symptoms included: (i) marginalization and concomitant degradation of abnormal strongly condensed chromatin (S5 Fig and Fig 7); (ii) extensive vacuolization connected with simultaneous formation of autolytic vacuoles (Fig 6F); (iii) formation of autophagy-like vesicles (Fig 6F); (iv) fragmentation of the cell nucleus (S5A and S5C Fig); and finally (v) extensive and irreversible degradation of the interior of the cell (Fig 7 and S7 Fig; Importantly, the degradation of the cell interior was not accompanied by a large scale inflammatory response, probably on account of the specificity of the plant material–the presence of cellular walls).

Similar morphological PCD criteria were distinguished in ultrastructural studies conducted on a model of the symbiotic sea anemone *Aiptasia sp*. [61]. Ultrastructural modeling of *V*. *faba* cell death showed likeness to the symptoms observed in both animals [62] and plants (among others [63–64]); the most typical additional manifestations included: (i) protoplast shrinkage (S5B Fig); (ii) condensed chromatin peripheral distribution in contact with the nuclear envelope membranes (S5C Fig); (iii) the appearance of irregularities in the cell nucleus shape, finally leading to degradation of the nucleus (Fig 7); (iv) formation of vesicular structures in the region of the nuclear envelope (S6C Fig); and (v) creation of multi-membrane myelin bodies (S6 Fig and Fig 7).

The characteristic co-occurrence of multi-membrane nuclear envelope sections with regions of chromatin with an exceptionally dense fibril packing seem to be a hallmark of the relatively early stages of (V/A) AL-PCD induced in *V*. *faba* root meristems (comp. [61]). This might reflect the formation of foci with particularly intensified biochemical processes, e.g. local accumulation of protein kinases. On the other hand, transition of the condensed chromatin to the nucleus periphery and the breakup of a nucleus into small fragments as well as its degradation, seem to be characteristic of final cell degradation (i.e. 'last step' [41–42,56]). The phenomenon of cell death is accompanied by proteolysis, often mediated by cysteine proteinases–caspases [65], while a particular role in the synthesis and accumulation of these proteins is ascribed to mitochondria [66]. The 'point of no return' can be reached through metabolic changes in the mitochondria; the launch of metabolic-morphological changes at the nuclear level means that the cell has already exceeded this point [42].

Analysis of the effect of salt and osmotic stress on the survival of theoretically immortal unicellular freshwater alga *Micrasterias denticulata* showed not only vacuolization and changes within Golgi and ER, but also extreme deformation of mitochondria [67]. Unexpectedly, we found no CF-induced morphological changes in the mitochondria (Fig 6D and S6A Fig). However, the presence or absence of symptoms within the mitochondria (and/or plastids) appears to depend on the type of agent inducing PCC. Accordingly, in a previous study using 2-aminopurine (as an inducer of PCC), changes were observed in both mitochondria and plastids (electron dense matrix with dilated invaginations of their internal membranes; TEM results [21]).

We found that in the described PCC induction scheme, the signaling cascade involved in the realization of the successive stages of V/A-type AL-PCD was exclusively transferred to cells adjacent to a dying cell, the process was slow, and was not intensive (as confirmed by the small scale of the phenomenon despite an almost total degradation of the cell from which the signaling cascade started; Fig 7; comp. [68,69]). Both the dying cell (until a certain moment) and the neighboring cell (all the time) continually attempted to counteract DNA damage through intensification of DNA repair processes (Rybaczek, in preparation).

We also described the formation of autophagosome-like structures (Fig 6F). Indeed, the formation of autophagosomes has been suggested in cell death in plants [70]. However, in our opinion, the appearance of real autophagosomes during AL-PCD in *V*. *faba* was not certain, and so requires further research. We previously showed that the activation of cell cycle checkpoints under HU-induced replication stress, overriding checkpoints during CF-induced PCC (under prolonged replication stress), was a multistage process requiring the participation of many proteins, and included: (i) detection of an abnormality (by sensory protein factors); (ii) transfer of a signal (through a signal transmission factors) and (iii) initiation of adequate responses (e.g. cell cycle arrest, DNA repair, etc.) *via* effector factors. Such a cellular response is highly complex, strictly controlled and usually very effective. However in PCC induction, i.e. during the accumulation of DNA damage, especially single- and double-stranded breaks, the cell needs to choose between survival and PCD initiation; in such a situation the latter is activated. Literature suggests that cells can enter the PCD pathway in both G1 and G2 phases of the cell cycle [71]. *V*. *faba* cells located in the G1 phase showed no signs of PCD [53], and their high number in the G1 phase could testify rather to blocking of the cell cycle in PCPI (Principal Control Point I). This may be part of a defense against PCD initiation, as is the case with blocking the transition of cancer cells in G2/M (PCPII; [72]) as well as with defense against the initiation of MC, however, this can occur only in dividing cells [13]. Entering into an endoreplication cycle may be another way to prevent PCD and the initiation of events leading to genomic instability [73].

ATR kinase is one of the sensory factors involved in the response associated with the activation of checkpoints. In turn, Brown and Baltimore [47] described that apoptosis can be caused by the elimination of ATR kinase, which prompts then to conclude that the accumulation of damaged DNA molecules can activate the pathway leading a cell to death. Smetana et al. [74], while observing E2F overexpression and CF-triggered ATM/ATR inhibition, and—on the basis of morphological criteria—revealed the capacity to undergo non-apoptotic death with paraptotic-like features in bleomycin-treated dividing tobacco BY-2 cells. This newly discovered kind of PCD was supported by revealing extensive vacuolization, vacuolar rupture and chromatin condensation, as well as a lack of apoptotic-type DNA fragmentation or sensitivity to caspase inhibitors.

Until recently, studies on PCD in plants most often used suspension culture models [5,54,74,75–77]. In 2011, however, a new classification of cell death was proposed, according to which 'autolytic' and 'non-autolytic' deaths were related solely to phenomena observed in intact plants but not in cell cultures [5]. Our study was conducted *in planta*, which gave the opportunity to interpret the results associated with the induction of (V/A) AL-PCD in the context of functioning of an entire plant, and certainly—of the a whole organ (root) or a specific zone in the root (i.e. meristematic zone).

The relatively small number of cells dying *via* (V/A) AL-PCD as a result of PCC induction (i.e. according to our experimental model) may be due to the internal heterogeneity of the meristem (reflecting lack of synchronization to entry into premature mitosis; comp. [21]), which should be considered in the context of the diverse metabolic statuses of different groups of cells that constitute the individual zones in the root. This heterogeneity makes it significantly more difficult to force more cells to enter the AL-PCD pathway, but at the same time it does not make it impossible to study cells that had already initiated this pathway.

We found that changes in the chromatin structure in the meristematic cells of *V*. *faba* were of a transient character. Seedlings incubated in water after a 24-h period of HU treatment and 8 h incubation in the HU/CF mixture, had restored mitotic activity in the tip zone after 12 h (Rybaczek, in preparation). The fates of the cells in which PCC induction occurred were also different. Some of them either restored mitotic divisions or enriched the pool of cells undergoing accelerated differentiation [21], while a small number–probably cells that had entered PCC from the S phase (S-PCC)–initiated a program that led to their elimination (*via* (V/A) AL-PCD, as shown in the present paper).

Most cellular reactions are reversible; proteins undergo phosphorylation and dephosphorylation, G proteins oscillate between a GTP-bound form and GDP-dependent state, second messengers are synthesized and degraded or released and sequestered, proteins are imported into or exported from a nucleus. Even proteolysis is reversible in a sense, because loss of degraded proteins can be compensated by their synthesis *de novo*. This does not mean of course that only reversible transformations occur in a cell. PCD, in both animal and plant cells, is an example of a cellular process that looks irreversible; especially if the changes in the cell nuclei during PCD reach 'the point beyond which the cell is irreversibly committed to die' [42,56]. The present study provides the first evidence that 'point of no return' can be the consequence of caffeine-induced genotoxic stress in HU-synchronized *V*. *faba* root meristems, and allows identification a specific type of cell death i.e. vacuolar/autolytic apoptotic-like PCD.

In conclusion, the current study has revealed that the cell death phenomenon in plants [8,30,46,53,55] is similar to that in animals [10,11] and human [77]. The results achieved in the introduced experimental model (triggering of cell death after PCC induction *in planta*) may serve as the basis for further stages of research aimed at identifying the mechanisms and signaling pathways responsible for triggering and conducting programmed cell death.

## Supporting Information

S1 Fig Root morphology, scheme of the experimental system and images after Feulgen staining. (A-C) Phenotypes of *Vicia faba* seedlings (A) control seedlings (untreated, incubated in water for 32 h); (B) seedlings treated with 2.5 mM hydroxyurea (HU) for 32 h; (C) seedlings synchronized with the use of 2.5 mM HU and then co-treated with 2.5 mM HU and 5 mM caffeine (CF) for additional 8 h. *Scale bar* in S1A Fig is 20 mm. (A-C) The frames placed in the bottom right corners show 1.5-cm root fragments (computer enlarged) that were subjected to further stages of experimental procedures. (A'-C') The schemes of the experiment. (A''-C'') Mitotic figures (anaphases) observed in the Feulgen-stained preparations from (A'') control seedlings, (B'') seedlings treated with HU for 32 h, (C'') seedlings pre-incubated with HU for 24 h and then transferred into the HU/CF. The anaphase seen in the image (A'') shows the correct morphology (phenotype A), asterisk (*) indicate only the occurrence of secondary constrictions that are not stained by Feulgen’s method. *Scale bar* in A'' = 10 μm is applied to all figures (from A'' to E'). (B'') Delicate aberrations indicated by an arrow, caused by the influence of HU (qualified neither to phenotype B [G2-PCC] nor to phenotype C [S-PCC], and rather closer to spontaneous aberrations, comp. [36]). (C'') The symptoms of premature chromosome condensation (PCC) during S-PCC-type anaphase represented by numerous fragmentations without chromatid-like pair elements (comp. [14]). (D) The formation of macronuclei was found significantly increased in comparison with the control. (E) Representative nuclei displaying signs of apoptosis-like programmed cell death (AL-PCD), i.e. interphase nuclei of the cells induced by the influence of CF first to PCC, and later to AL-PCD. (E') Chromosome segregation defects as a consequence of CF-induced G2-type PCC.
(TIF)

S2 Fig Qualitative assessment of DNA fragmentation. The fragmentation of genomic DNA was studied in *Vicia faba* root meristem cells exposed to hydroxyurea (HU) for 32 h (lane 2) as well as during the induction of premature chromosome condensation (PCC, lane 3), in comparison either with control (lane 1) or DNA marker (1,500–6,000 bp, lane M). DNA was stained with ethidium bromide (EB) and separated DNA samples were visualized under UV light.
(TIF)

S3 Fig Micrographic pictures showing acridine orange (AO) and ethidium bromide (EB) staining in *Vicia faba* roots. Comparison between (A-A'') the control roots, (B-B'') the roots treated with hydroxyurea (HU) for 32 h, (C-C'') the roots treated with HU for 24 h and then co-treated with HU/caffeine (CF) for the next 8 h. (B-B'') Arrows were used to mark the places, in which HU-treated roots undergo a distinct widening forming well visible protuberance. In the place of the protuberances occurrence, one could observe the accumulation of dead cells (B-B''). Broken lines were used to mark the outline of the protuberances (B-B''). The occurrence of a protuberance was limited to the zone of dividing cells (B-B''). (C-C'') Two-headed arrows presents the quiescent centers (QCs) of roots subjected to PCC (HU/CF-treated). QC shows yellow-orange fluorescence that indicates dying and dead cells in it. Three-headed arrows in the picture (C-C'') indicate the accumulation of cells with yellow-orange fluorescence (dying) but observed in the meristem region. *Scale bar* = 1 mm.
(TIF)

S4 Fig Electron micrographs of *Vicia faba* root meristem cells. (A) control (32-h incubation in water); (B) hydroxyurea-treated (32-h); (C) hydroxyurea (HU) synchronized for 24 h and then HU and caffeine (CF) co-treated (for successive 8 h; total incubation time: 32 h). The cells presented in figures (A) and (B) show no significant differences, apart from deposits presence in vacuoles after treatment with HU (B, marked with an asterisk). The vacuoles of the control series do not contain any deposits (A). (C) Symptoms of early events of apoptosis-like programmed cell death (AL-PCD) induced under the influence of HU/CF: irregular chromatin condensation, invagination of nuclear envelope, presence of enzymatically predigested or digested organelles in lytic vacuole (structures marked with asterisk in figure C). *c* cytoplasm, *cw* cell wall, *lv* lytic vacuole, *m* mitochondrion, *n* nucleus, *ne* nuclear envelope, *NeI* invagination of nuclear envelope; *no* nucleolus, *nov* nucleolus vacuole, *p* plastid, *pd* plasmodesmata, *s* starch, *v* vacuole. *Scale bar* = 5 μm.
(TIF)

S5 Fig Symptoms of apoptosis-like programmed cell death (AL-PCD) induced by 24-h treatment with hydroxyurea (HU) and co-treatment with the mixture of HU and caffeine (CF) for successive 8 h. (A-C) Successive pictures presenting a probable sequence of events connected with a gradual intensification of AL-PCD symptoms in *Vicia faba* root meristem cells previously induced to PCC. (A) Symptoms accompanying the successive stadia of cellular nucleus fragmentation: irregular chromatin condensation; accumulation of strongly condensed chromatic aggregates near the nuclear envelope (marked with asterisks); the formation of myelin-like structures (sometimes strongly developed and multi-layer, accumulating either near plasmalemma or in regions connected with nuclear envelope [marked with double arrow heads]); degradation of organelles inside lytic vacuoles (marked with arrows). (B) Progressing development of myelin-like structures (*mls*) and distinct shrinkage of protoplast (direction of protoplast shrinkage is marked with arrows). (C) Progressing degradation of nuclear chromatin connected with displacement of its super-condensed form towards nuclear envelope and the formation of brightening in the central part on nucleus; further formation of multi-membrane structures in the regions connected with plasmalemma (marked with asterisks) or connected with the nuclear envelope (marked with symbol: *mne*); the formation of autophagosome-like structures (an arrow). *c* cytoplasm, *cw* cell wall, *dch* dense chromatin, *lv* lytic vacuole, *m* mitochondrion, *mls* multilamellar structure; *mne* multilamellar nuclear envelope; *n* nucleus, *no* nucleolus, *p* plastid, *pd* plasmodesmata, *s* starch; *sdch* supercondensed dense chromatin. *Scale bar* = 5 μm.
(TIF)

S6 Fig Diversity of generated multi-membrane structures and structures with cloudy morphology during apoptosis-like programmed cell death (AL-PCD) induced by the treatment with hydroxyurea (HU) for 24 h and co-treatment with the mixture of HU and caffeine (CF) for successive 8 h. Structures with cloudy morphology are marked with asterisks (A-C). Myelin structures are marked as '*mls*' if these were localized in cytoplasm (B) or '*mne*' if were connected localization-wise with the external nucleus envelope layers (B). Multi-membrane structures touching plasmalemma are marked with arrows (B-C). *c* cytoplasm, *cl* cludy-like morphology structure; *cw* cell wall, *dch* dense chromatin, *lv* lytic vacuole, *m* mitochondrion, *mls* multilamellar structure; *mne* multilamellar nuclear envelope; *n* nucleus. *Scale bar* = 5 μm.
(TIF)

S7 Fig Advanced stadium of the cellular nucleus fragmentation in the course of apoptosis-like programmed cell death (AL-PCD) induced by 24-h treatment with hydroxyurea (HU) and co-treatment with the mixture of HU and caffeine (CF) for successive 8 h. (A) Fragmented nucleus containing strongly condensed chromatin is suspended in the space of electron lucent cell with partially digested organelles pushed onto the cell periphery, i.e. near plasmalemma. In the top part of a cell, one can observe initial stadia of protoplast shrinkage. The plasma membrane exhibits multilamellar morphology (black regions visible on the cell periphery). (B) Progressing chromatin condensation and further nucleus fragmentation and marginalization. The interior of almost whole cell is filled by an enormous lytic vacuole. Cellular organelles, digested to a large extent, are pushed towards extreme cell regions. Asterisks (*) indicate the electron transparent spaces that are: (1) localized inside a nucleus; (2) border the masses of supercondensed chromatin and (3) are still surrounded by a double layer of nuclear envelope (A-B). *lv* lytic vacuole, *n* nucleus. *Scale bar* = 5 μm.
(TIF)
