# Supplementary figures and images for: Caffeine-Induced Premature Chromosome Condensation Results in the Apoptosis-Like Programmed Cell Death in Root Meristems of Vicia faba
Source: PLoS One. 2015 Nov 6;10(11):e0142307. doi: 10.1371/journal.pone.0142307 (PMC4636323; doi:10.1371/journal.pone.0142307)

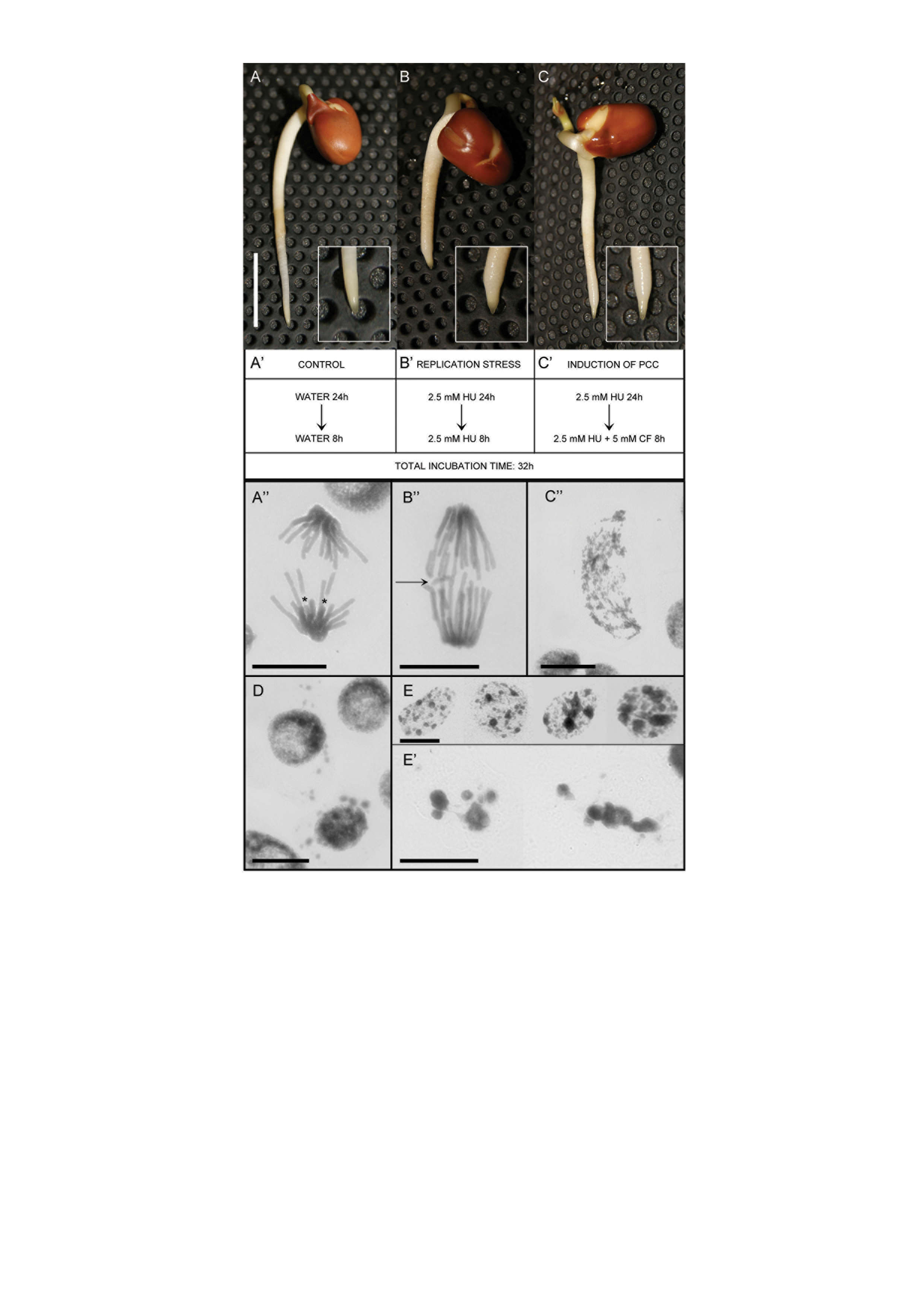

Supplement: S1 Fig — (A-C) Phenotypes of Vicia faba seedlings (A) control seedlings (untreated, incubated in water for 32 h); (B) seedlings treated with 2.5 mM hydroxyurea (HU) for 32 h; (C) seedlings synchronized with the use of 2.5 mM HU and then co-treated with 2.5 mM HU and 5 mM caffeine (CF) for additional 8 h. Scale bar in S1A Fig is 20 mm. (A-C) The frames placed in the bottom right corners show 1.5-cm root fragments (computer enlarged) that were subjected to further stages of experimental procedures. (A'-C') The schemes of the experiment. (A''-C'') Mitotic figures (anaphases) observed in the Feulgen-stained preparations from (A'') control seedlings, (B'') seedlings treated with HU for 32 h, (C'') seedlings pre-incubated with HU for 24 h and then transferred into the HU/CF. The anaphase seen in the image (A'') shows the correct morphology (phenotype A), asterisk (*) indicate only the occurrence of secondary constrictions that are not stained by Feulgen’s method. Scale bar in A'' = 10 μm is applied to all figures (from A'' to E'). (B'') Delicate aberrations indicated by an arrow, caused by the influence of HU (qualified neither to phenotype B [G2-PCC] nor to phenotype C [S-PCC], and rather closer to spontaneous aberrations, comp. [36]). (C'') The symptoms of premature chromosome condensation (PCC) during S-PCC-type anaphase represented by numerous fragmentations without chromatid-like pair elements (comp. [14]). (D) The formation of macronuclei was found significantly increased in comparison with the control. (E) Representative nuclei displaying signs of apoptosis-like programmed cell death (AL-PCD), i.e. interphase nuclei of the cells induced by the influence of CF first to PCC, and later to AL-PCD. (E') Chromosome segregation defects as a consequence of CF-induced G2-type PCC. (TIF) [file pone.0142307.s001.tif]

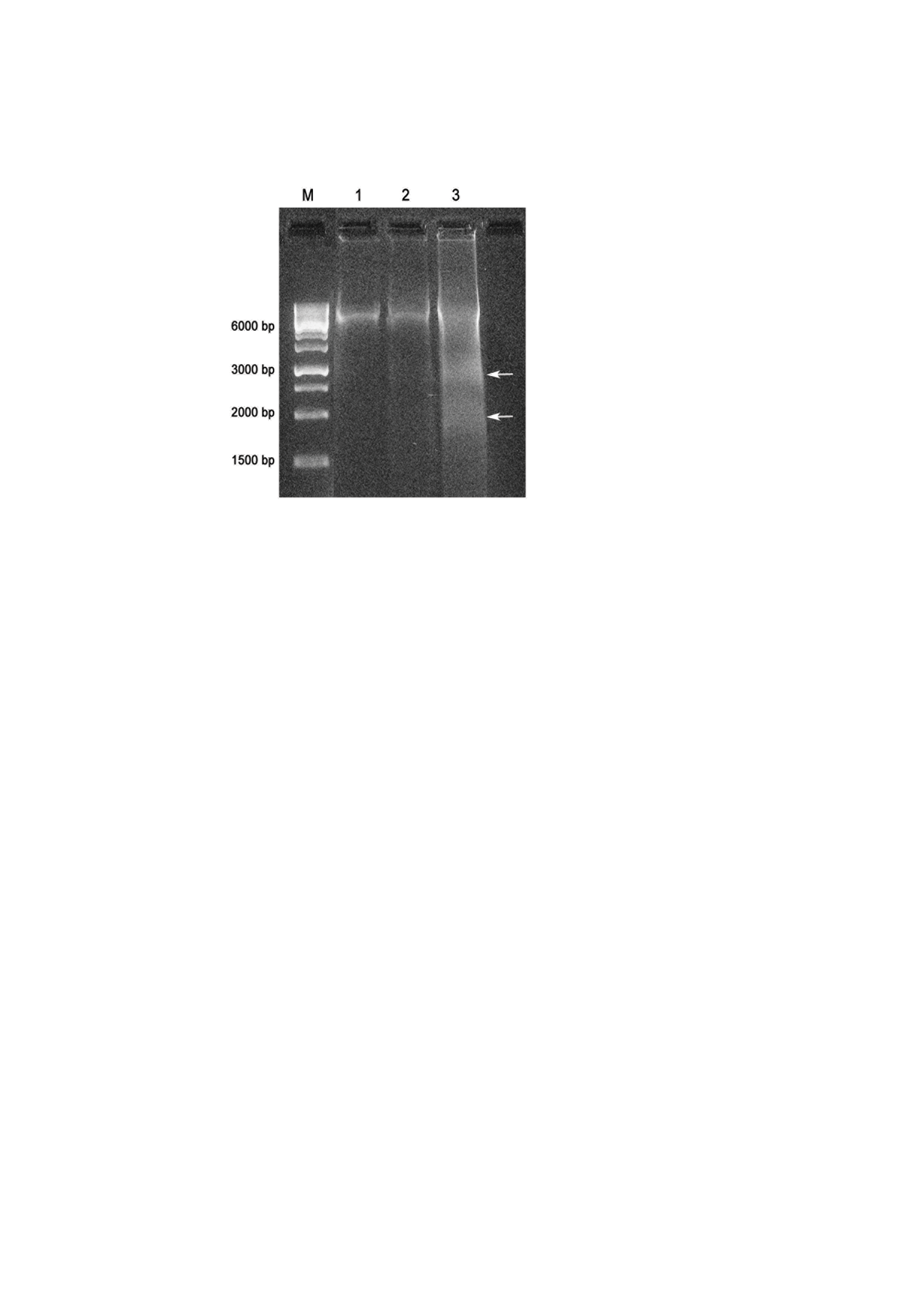

Supplement: S2 Fig — The fragmentation of genomic DNA was studied in Vicia faba root meristem cells exposed to hydroxyurea (HU) for 32 h (lane 2) as well as during the induction of premature chromosome condensation (PCC, lane 3), in comparison either with control (lane 1) or DNA marker (1,500–6,000 bp, lane M). DNA was stained with ethidium bromide (EB) and separated DNA samples were visualized under UV light. (TIF) [file pone.0142307.s002.tif]

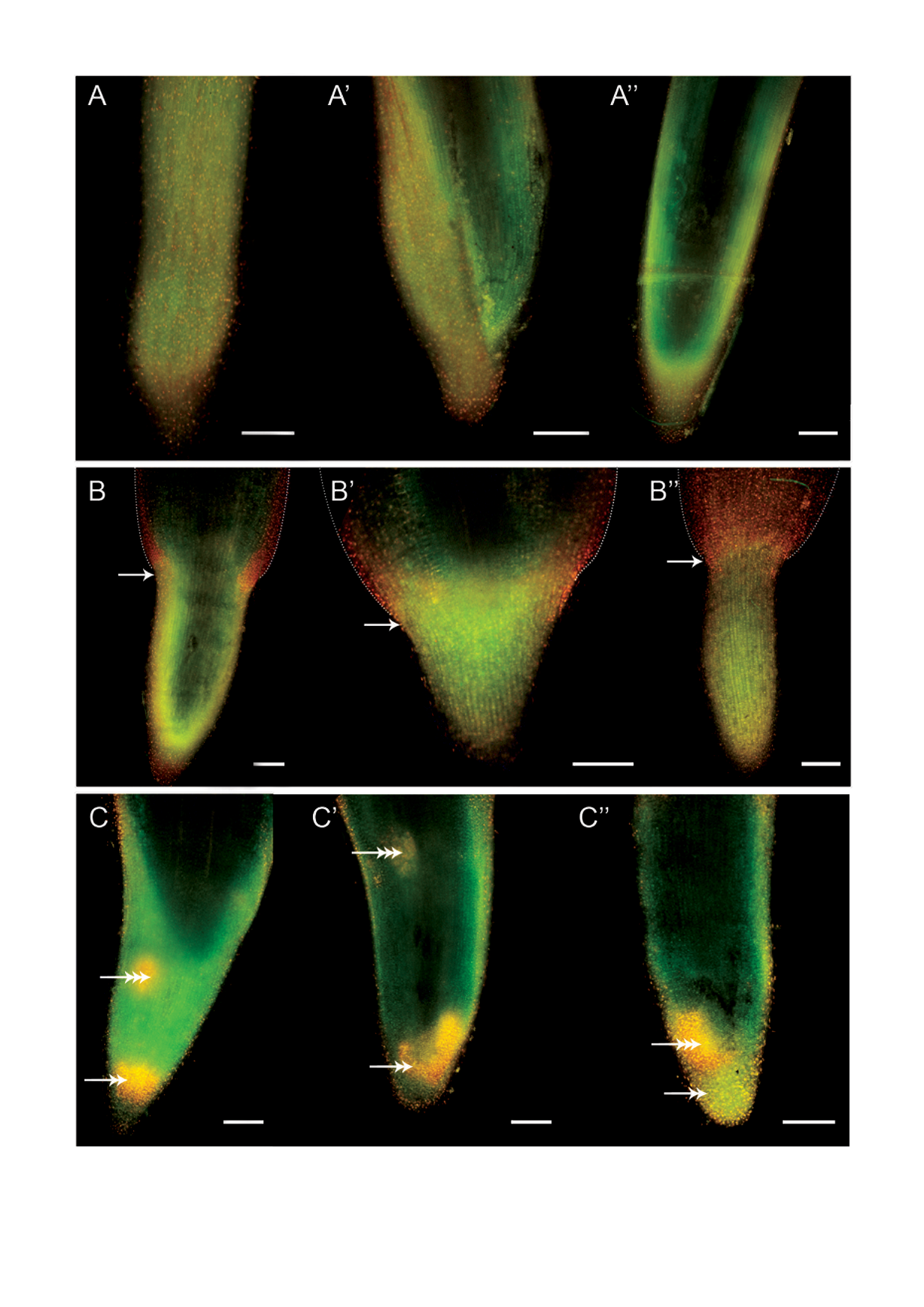

Supplement: S3 Fig — Comparison between (A-A'') the control roots, (B-B'') the roots treated with hydroxyurea (HU) for 32 h, (C-C'') the roots treated with HU for 24 h and then co-treated with HU/caffeine (CF) for the next 8 h. (B-B'') Arrows were used to mark the places, in which HU-treated roots undergo a distinct widening forming well visible protuberance. In the place of the protuberances occurrence, one could observe the accumulation of dead cells (B-B''). Broken lines were used to mark the outline of the protuberances (B-B''). The occurrence of a protuberance was limited to the zone of dividing cells (B-B''). (C-C'') Two-headed arrows presents the quiescent centers (QCs) of roots subjected to PCC (HU/CF-treated). QC shows yellow-orange fluorescence that indicates dying and dead cells in it. Three-headed arrows in the picture (C-C'') indicate the accumulation of cells with yellow-orange fluorescence (dying) but observed in the meristem region. Scale bar = 1 mm. (TIF) [file pone.0142307.s003.tif]

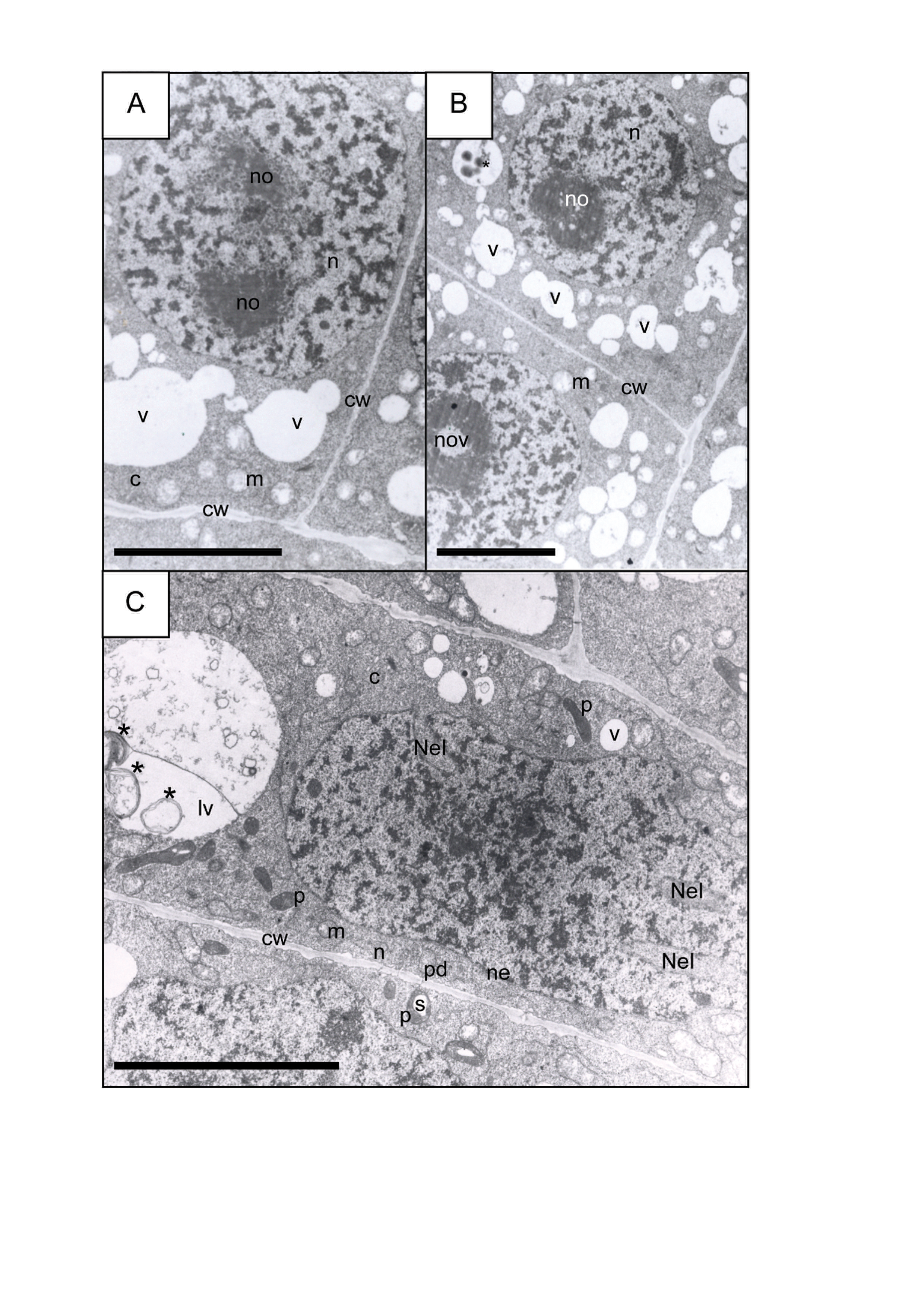

Supplement: S4 Fig — (A) control (32-h incubation in water); (B) hydroxyurea-treated (32-h); (C) hydroxyurea (HU) synchronized for 24 h and then HU and caffeine (CF) co-treated (for successive 8 h; total incubation time: 32 h). The cells presented in figures (A) and (B) show no significant differences, apart from deposits presence in vacuoles after treatment with HU (B, marked with an asterisk). The vacuoles of the control series do not contain any deposits (A). (C) Symptoms of early events of apoptosis-like programmed cell death (AL-PCD) induced under the influence of HU/CF: irregular chromatin condensation, invagination of nuclear envelope, presence of enzymatically predigested or digested organelles in lytic vacuole (structures marked with asterisk in figure C). c cytoplasm, cw cell wall, lv lytic vacuole, m mitochondrion, n nucleus, ne nuclear envelope, NeI invagination of nuclear envelope; no nucleolus, nov nucleolus vacuole, p plastid, pd plasmodesmata, s starch, v vacuole. Scale bar = 5 μm. (TIF) [file pone.0142307.s004.tif]

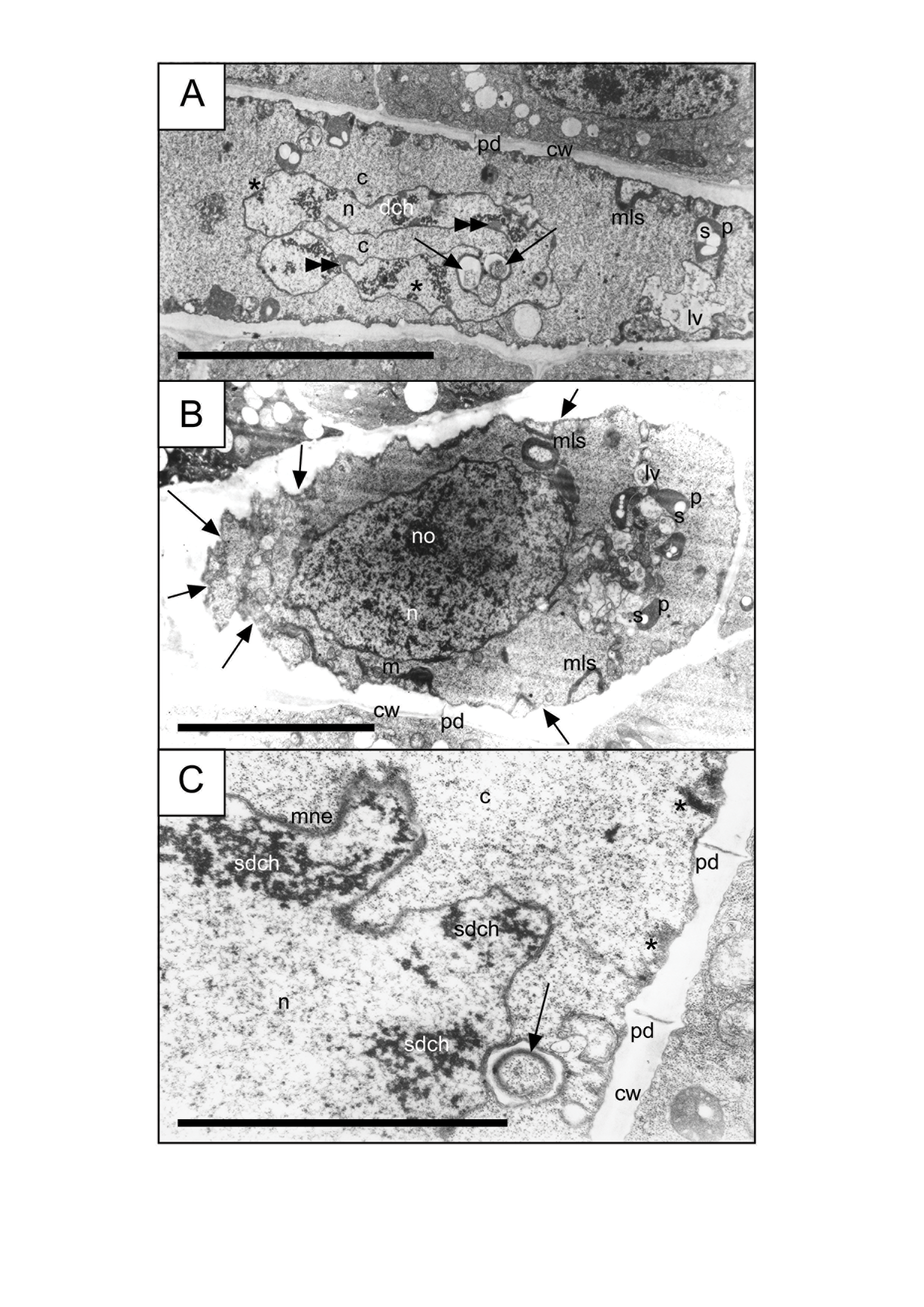

Supplement: S5 Fig — (A-C) Successive pictures presenting a probable sequence of events connected with a gradual intensification of AL-PCD symptoms in Vicia faba root meristem cells previously induced to PCC. (A) Symptoms accompanying the successive stadia of cellular nucleus fragmentation: irregular chromatin condensation; accumulation of strongly condensed chromatic aggregates near the nuclear envelope (marked with asterisks); the formation of myelin-like structures (sometimes strongly developed and multi-layer, accumulating either near plasmalemma or in regions connected with nuclear envelope [marked with double arrow heads]); degradation of organelles inside lytic vacuoles (marked with arrows). (B) Progressing development of myelin-like structures (mls) and distinct shrinkage of protoplast (direction of protoplast shrinkage is marked with arrows). (C) Progressing degradation of nuclear chromatin connected with displacement of its super-condensed form towards nuclear envelope and the formation of brightening in the central part on nucleus; further formation of multi-membrane structures in the regions connected with plasmalemma (marked with asterisks) or connected with the nuclear envelope (marked with symbol: mne); the formation of autophagosome-like structures (an arrow). c cytoplasm, cw cell wall, dch dense chromatin, lv lytic vacuole, m mitochondrion, mls multilamellar structure; mne multilamellar nuclear envelope; n nucleus, no nucleolus, p plastid, pd plasmodesmata, s starch; sdch supercondensed dense chromatin. Scale bar = 5 μm. (TIF) [file pone.0142307.s005.tif]

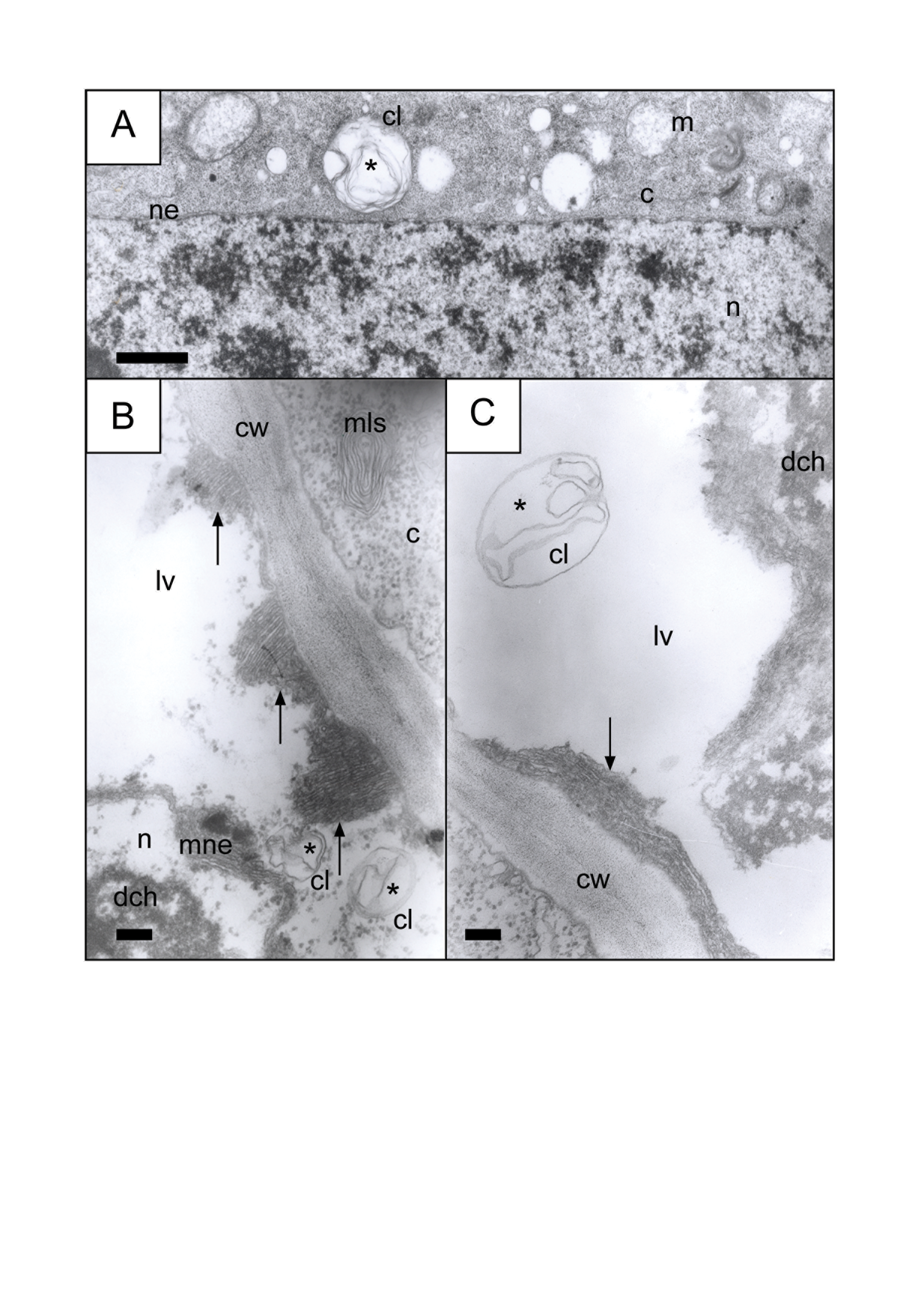

Supplement: S6 Fig — Structures with cloudy morphology are marked with asterisks (A-C). Myelin structures are marked as 'mls' if these were localized in cytoplasm (B) or 'mne' if were connected localization-wise with the external nucleus envelope layers (B). Multi-membrane structures touching plasmalemma are marked with arrows (B-C). c cytoplasm, cl cludy-like morphology structure; cw cell wall, dch dense chromatin, lv lytic vacuole, m mitochondrion, mls multilamellar structure; mne multilamellar nuclear envelope; n nucleus. Scale bar = 5 μm. (TIF) [file pone.0142307.s006.tif]

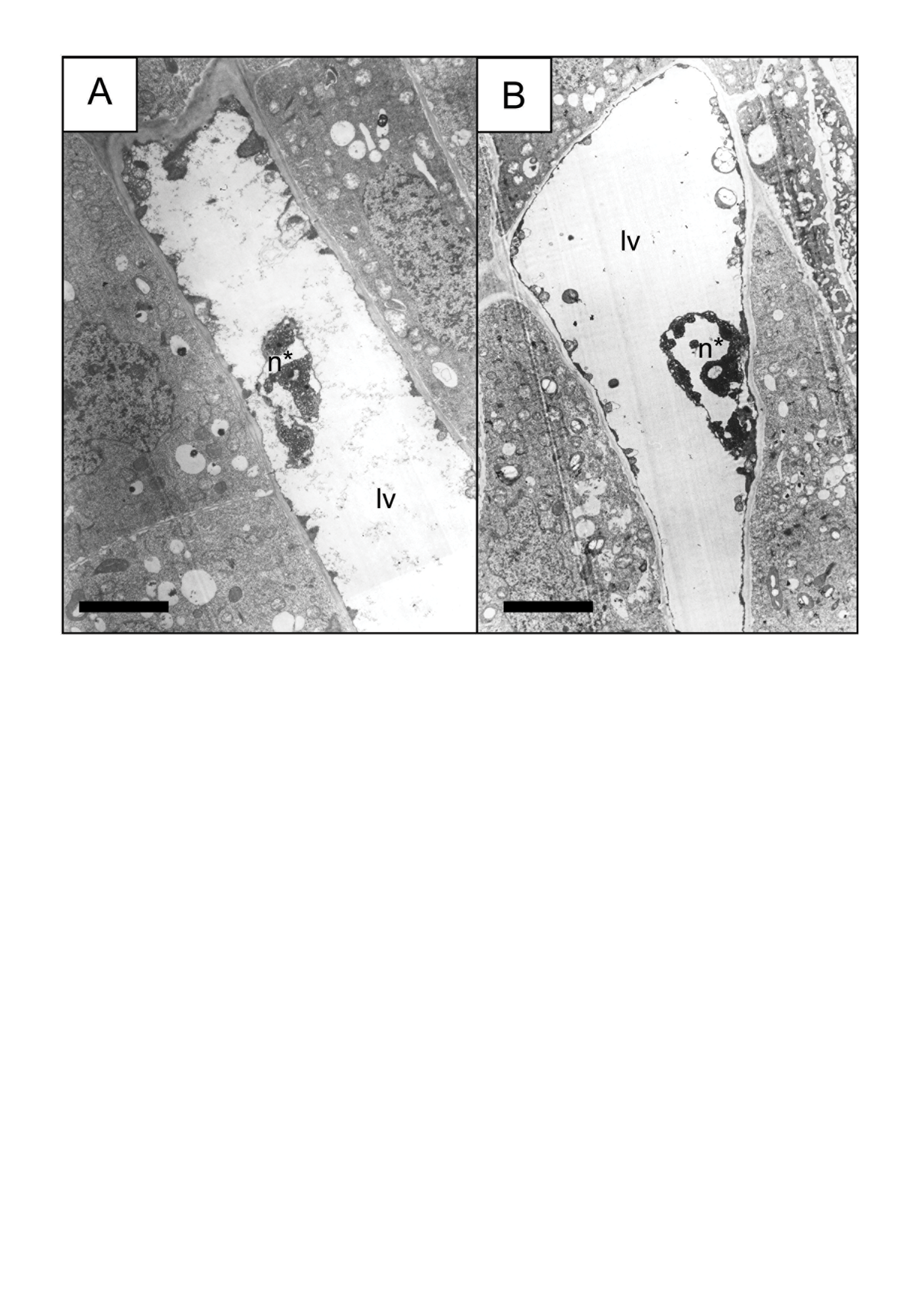

Supplement: S7 Fig — (A) Fragmented nucleus containing strongly condensed chromatin is suspended in the space of electron lucent cell with partially digested organelles pushed onto the cell periphery, i.e. near plasmalemma. In the top part of a cell, one can observe initial stadia of protoplast shrinkage. The plasma membrane exhibits multilamellar morphology (black regions visible on the cell periphery). (B) Progressing chromatin condensation and further nucleus fragmentation and marginalization. The interior of almost whole cell is filled by an enormous lytic vacuole. Cellular organelles, digested to a large extent, are pushed towards extreme cell regions. Asterisks (*) indicate the electron transparent spaces that are: (1) localized inside a nucleus; (2) border the masses of supercondensed chromatin and (3) are still surrounded by a double layer of nuclear envelope (A-B). lv lytic vacuole, n nucleus. Scale bar = 5 μm. (TIF) [file pone.0142307.s007.tif]
